# TTC3 contributes to TGF-β_1_-induced epithelial−mesenchymal transition and myofibroblast differentiation, potentially through SMURF2 ubiquitylation and degradation

**DOI:** 10.1038/s41419-019-1308-8

**Published:** 2019-01-29

**Authors:** June-Hyung Kim, Sangwoo Ham, Yunjong Lee, Gee Young Suh, Yun-Song Lee

**Affiliations:** 1 Division of Pharmacology, Department of Molecular & Cellular Biology, Sungkyunkwan University School of Medicine, Samsung Biomedical Research Institute, Suwon, Republic of Korea; 2 Department of Critical Care Medicine in Samsung Medical Center, Sungkyunkwan University School of Medicine, Seoul, Republic of Korea

## Abstract

Transforming growth factor-β (TGF-β) acts as a key cytokine in epithelial−mesenchymal transition (EMT) and myofibroblast differentiation, which are important for normal tissue repair and fibrotic diseases. Ubiquitylation and proteasomal degradation of TGF-β signaling proteins acts as a regulatory mechanism for the precise control of TGF-β signaling. SMAD-specific ubiquitin E3 ligase (SMAD ubiquitination regulatory factor 2, SMURF2) controls TGF-β signaling proteins including the TGF-β receptor (TGFR) and SMAD2/3. Here, we report that tetratricopeptide repeat domain 3 (TTC3), a ubiquitin E3 ligase, positively regulates TGF-β_1_-induced EMT and myofibroblast differentiation, through inducing ubiquitylation and proteasomal degradation of SMURF2. In human bronchial epithelial cells (BEAS-2B) and normal human lung fibroblasts, TTC3 knockdown suppressed TGF-β_1_-induced EMT and myofibroblast differentiation, respectively. Similarly, when TTC3 expression was suppressed, the TGF-β_1_-stimulated elevation of p-SMAD2, SMAD2, p-SMAD3, and SMAD3 were inhibited. In contrast, overexpression of TTC3 caused both EMT and myofibroblast differentiation in the absence of TGF-β_1_ treatment. TGF-β_1_ reduced SMURF2 levels and TTC3 overexpression led to a further decrease in SMURF2 levels, while TTC3 knockdown inhibited TGF-β_1_-induced SMURF2 reduction. In cell and in vitro ubiquitylation assays demonstrated TTC3-mediated SMURF2 ubiquitylation, and coimmunoprecipitation assays established the binding between SMURF2 and TTC3. TGF-β_1_-induced TTC3 expression was inhibited by the knockdown of SMAD2 and SMAD3. Finally, *Ttc3* mRNA levels were significantly increased and Smurf2 protein levels were significantly decreased in the lungs of mice treated with bleomycin as compared with the lungs of control mice. Collectively, these data suggest that TTC3 may contribute to TGF-β_1_-induced EMT and myofibroblast differentiation, potentially through SMURF2 ubiquitylation/proteasomal degradation and subsequent inhibition of SMURF2-mediated suppression of SMAD2 and SMAD3, which in turn induces TTC3 expression.

## Introduction

The epithelial−mesenchymal transition (EMT) is observed not only in physiological processes such as development and wound healing, but also in pathological processes such as fibrotic diseases and cancer metastasis^1,2^. In the EMT process, epithelial cells lose polarity and have enhanced migratory capacity, invasiveness, and increased production of extracellular matrix (ECM) components, together with a downregulation of epithelial signature genes including E-cadherin and zona occludens-1 (ZO-1), and an upregulation of genes characterizing mesenchymal cells including N-cadherin and vimentin^3^. TGF-β is a potent inducer of EMT, and EMT caused by deregulated repair processes is suggested to be responsible for pathological organ fibrosis^4,5^.

Similar to EMT, TGF-β potently induces myofibroblast differentiation in normal wound healing and fibrotic diseases. Myofibroblasts have features of both fibroblasts and smooth muscle cells, which proficiently produce ECM proteins and have contractile properties given their expression of α-smooth muscle actin (α-SMA)^6^. Typically, there is a regression and disappearance of myofibroblasts by apoptosis during normal wound healing, and the perpetual existence of myofibroblasts may be the cause of some fibrotic diseases. Among multiple origins, resident fibroblasts and mesenchymal cells derived from epithelial cells during EMT are important sources of myofibroblasts that are involved in pathological fibrosis such as pulmonary fibrosis^7^.

The canonical pathway of TGF-β signaling consists of TGF-β receptors (TGFRs) and receptor-regulated SMADs (R-SMADs)^8^. TGF-β binds to a heteromeric receptor complex consisting of two TGFR1 and two TGFR2. Phosphorylation of TGFR1 by TGFR2 permits the binding and phosphorylation of R-SMADs (SMAD2 and SMAD3). Phosphorylated R-SMADs form a heteromeric complex with SMAD4, and the complex translocates into the nucleus where the complex regulates the expression of TGF-β-inducible genes. TGF-β signaling is regulated by various inhibitory mechanisms including ubiquitylation and proteasomal degradation of the associated signaling molecules^9^. As a part of negative feedback, SMAD7 induced by the activated SMAD complexes acts as a scaffold to recruit SMAD ubiquitin E3 ligase 2 (SMURF2), a HECT (homologous to the E6-AP carboxyl terminus)-type ubiquitin E3 ligase, which facilitates TGFR degradation, thereby attenuating TGF-β signaling^10^. In addition, SMURF2 causes the degradative polyubiquitylation of SMAD2^11,12^ and SMAD3^13^ and multiple monoubiquitylation of SMAD3, inhibiting the formation of SMAD3 complexes^14^. Hence, SMURF2 is considered one of the key TGF-β regulatory molecules.

Tetratricopeptide repeat domain 3 (TTC3), whose gene is located in the Down syndrome critical region^15^, was found to act as a ubiquitin E3 ligase for Akt^16^. TTC3 was involved in cigarette smoking-induced cell death^17^, neuronal differentiation^18,19^, and asymmetric cell division in cancer cells^20^. However, to our knowledge, the involvement of TTC3 in other signaling pathways and other pathophysiological processes has not been reported. Here, we report a novel finding that TTC3 contributes to TGF-β-induced EMT and myofibroblast differentiation in a feedforward fashion. This potentially occurs through TTC3 inducing the ubiquitylation and proteasomal degradation of SMURF2, which elevates SMAD2 and SMAD3, and, in turn, induces TTC3 expression.

## Materials and methods

Detailed information is available in Supplemental Materials and Methods.

Normal human lung fibroblasts (NHLFs) were purchased from Lonza (Walkersville, MD, USA). BEAS-2B cells and HEK293 cells were purchased from Korea Cell Line Bank (Seoul, Korea). Human TGF-β_1_ was obtained from R&D Systems (Minneapolis, MN, USA) and other chemicals were purchased from Sigma (St. Louis, MO, USA). Antibodies were purchased from the following companies: anti-E-cadherin, anti-zona occuludens 1 (ZO-1), anti-N-cadherin, anti-vimentin, anti-SMAD2, anti-SMAD3, anti-phospho-SMAD2 (Ser465/467), anti-phospho-SMAD3 (Ser423/425), anti-SMURF2, horse radish peroxidase (HRP)-conjugated anti-rabbit IgG and anti-mouse IgG (Cell Signaling, Beverly, MA, USA), anti-TTC3, anti-hemagglutinin (HA), anti-DYK (FLAG), and anti-Myc antibodies (Sigma), anti-α-SMA antibody (Abcam, Cambridge, MA, USA), anti-ubiquitin antibody (Santa Cruz Biotechnology, Santa Cruz, CA, USA), and anti-GAPDH antibody (AbFrontier, Seoul, Korea). Anti-DYK (M2) and anti-Myc agarose beads were purchased from Sigma and Thermo Scientific (Rockford, IL, USA), respectively. Detailed information about antibodies was given in Supplemental Table 1. Reagents including E1 (UBE1), E2 (UBE2E3), and the reaction buffer for the in vitro ubiquitylation assay were purchased from Boston Biochem (Cambridge, MA, USA).

### Plasmids

DYK-tagged wild-type pCMV6-TTC3 (WT-TTC3/DYK) was purchased from GenScript (Clone OHu03292, Piscataway, NJ, USA), and mutant TTC3 (ΔRF-TTC3/DYK) was generated by deleting the RING motif (1956Cys to 1997Gln, +5868 to +5993 bps), as previously described^16^. The wild-type SMURF2/Myc plasmid was obtained from Addgene (#13678, deposited by Ying Zhang, Cambridge, MA, USA). In order to observe K48-linked SMURF2 ubiquitylation, we used HA-tagged wild-type ubiquitin (WT-Ubi/HA), K48R ubiquitin (K48R-Ubi/HA; Lys48 replaced with Arg), K48 ubiquitin (K48-Ubi/HA; all Lys residues replaced with Arg except K48), and KO ubiquitin (KO-Ubi/HA; all Lys residues replaced with Arg) plasmids with the same backbone we used previously^17^.

### Cell culture and transfection

NHLFs (passages 6−10) and BEAS-2B cells were grown in Dulbecco’s modified Eagle’s medium (DMEM) and RPMI1640 containing 10% fetal bovine serum (FBS) (Gibco, Grand Island, NY, USA). For TGF-β_1_ treatment, NHLFs and BEAS-2B cells were washed three times with phosphate-buffered saline (PBS) and incubated overnight with fresh serum-free medium, to exclude the potential influences of serum factors. The following day, the cells were washed three times with PBS and grown with fresh serum-free DMEM containing 10 ng/ml TGF-β_1_^21^. The transfection of plasmids into NHLFs and BEAS-2B cells was performed as previously described with a slight modification^17^. Briefly, at 70% confluence in a 10 cm culture dish, the medium was replaced with fresh DMEM or RPMI containing 10% FBS, and transient transfection was performed using FuGENE HD transfection reagent (Promega, San Luis Obispo, CA, USA), according to the manufacturer’s instructions. For TGF-β_1_ treatment, transfected cells were deprived of serum overnight 1 day after transfection.

### RNA interference

TTC3 siRNA targeting the coding region of *TTC3* (TTC3 siRNA1, On-TARGETplus) and control scrambled siRNA were purchased from Dharmacon (Lafayette, CO, USA). TTC3 siRNA targeting the 3′-untranslated region (UTR) of *TTC3* was synthesized (TTC3 siRNA2, Bioneer, Daejeon, Korea). After confirming the effects of both TTC3 siRNAs, TTC3 siRNA1 was used in the rest of experiments. One hundred picomoles of control and target-specific siRNAs were transfected into BEAS-2B cells and NHLFs at 70% confluence in a 10 cm culture dish using RNAiMAX (Invitrogen, Carlsbad, CA, USA) according to the manufacturer’s instructions. One day after transfection, transfected cells were deprived of serum overnight for TGF-β_1_ treatment.

### Western blot analysis

At the indicated times, cells were harvested and lysed in a lysis buffer (Cell Signaling, MA, USA) containing protease and phosphatase inhibitor cocktails (Sigma). Following centrifugation at 18,000 × *g* at 4 °C for 20 min, proteins in supernatants were separated by SDS-PAGE, and transferred onto a nitrocellulose membrane (PanReac AppliChem, Darmstadt, Germany). Membranes were blocked with 5% skim milk for 1 h at room temperature, and incubated overnight with primary antibody diluted in 0.5% Tween-20 in PBS (PBS-T) (anti-TTC3 at 1:100; all other antibodies at 1:1000) at 4 °C. After washing with PBS-T, membranes were then incubated with HRP-conjugated secondary antibodies (1:5000). Protein bands were visualized using ECL reagents (Amersham, Piscataway, NJ, USA) and X-ray films (Agfa, Mortsel, Belgium). Images were captured with a scanner (Epson, Tokyo, Japan), and image densities were quantified with ImageJ (https://imagej.net).

### In cell assay of SMURF2 ubiquitylation

TTC3-mediated SMURF2 ubiquitylation was detected in HEK293 cells using polyethylenimine (PEI, Polyscience, Niles, IL, USA) as a transfection reagent at a 1:3 ratio of DNA to PEI. HEK293 cells at 70% confluence in a 10 cm culture dish were transfected with the TTC3/DYK plasmid (3 μg) on day 1, and then transfected with SMURF2/Myc (2 μg) and Ubi/HA (2 μg) plasmids on day 2. After overnight incubation, the cells were treated with 5 μM MG132. On day 4, the cells were lysed by sonication in 500 μl of Brij lysis buffer (10 mM Tris pH 7.5, 150 mM NaCl, 2.5 mM 2,2',2'',2'''-(ethane-1,2-diyldinitrilo) tetraacetic acid, 0.875% Brij 97, 0.125% NP40) containing protease and phosphatase inhibitor cocktails. After centrifugation at 18,000 × *g* at 4 °C for 20 min, SMURF2 was immunoprecipitated overnight with 20 μl of anti-Myc antibody-conjugated agarose beads at 4 °C. After the beads were washed five times with Brij lysis buffer, bound SMURF2 was eluted by boiling in 5× Laemmli sample buffer and ubiquitylated SMURF2 was detected with an anti-HA antibody (1:1000).

### In vitro assay of SMURF2 ubiquitylation

In vitro assay of ubiquitylation of SMURF2 was performed with 6×-His-tagged recombinant C-terminal wild-type TTC3 (WT-C-TTC3; Ile1540 to Arg2025), which were produced in Sf9 cells using the Bac-to-Bac Baculovirus expression system (Invitrogen), as described previously with slight modifications^16^. Briefly, WT-C-TTC3 was subcloned into pFastBac HT-B using pCMV6-WT-TTC3/DYK vector as a template, and DH10Bac *Escherichia coli* were transformed with the plasmids to generate baculoviruses. After 60 h of infection with passage 3 baculoviruses, Sf-9 cells were lysed in a buffer (50 mM NaH_2_PO_4_, pH 8.0, 0.5 M NaCl, and 10 mM imidazole) by three freeze-thaw cycles. After centrifugation at 3,000 × *g* at 4 °C for 15 min, recombinant TTC3 proteins were bound overnight to nickel-chelating resin (Life Technologies, Carlsbad, CA, USA) at 4 °C. The resin was washed with a lysis buffer containing 20 mM imidazole and 0.5 M LiCl, and the recombinant proteins were eluted with an elution buffer (lysis buffer containing 0.5 M imidazole, pH 8.0). In vitro ubiquitylation of SMURF2 was performed by incubating 300 ng DYK-tagged recombinant human SMURF2 (Abcam), 1 µg recombinant TTC3, 0.1 µg human E1 (UBE1), and 1 µg human E2 (UBE2E3) in 40 µl of reaction buffer for 4 h at 30 °C. The reaction mixtures were then heated in 5× Laemmli sample buffer at 95 °C for 5 min, and western blots were performed with an anti-ubiquitin antibody (1:1000).

### Coimmunoprecipitation

Binding between TTC3 and SMURF2 was assessed by reciprocal coimmunoprecipitation analysis. BEAS-2B cells were transfected with SMURF2/Myc or TTC3/DYK plasmids to detect the binding of endogenous TTC3 or SMURF2, respectively. After transfection, the cells were washed on day 1 and incubated without serum. On day 2, TGF-β_1_ was added at a concentration of 10 ng/ml, and the cells were harvested on day 3. MG132 at a concentration of 5 μM was added 12 h before harvesting the cells. After lysis in the Brij lysis buffer, cell lysates were centrifuged at 18,000 × *g* at 4 °C for 20 min. A total of 3.5 mg proteins was incubated with 60 μl of anti-Myc or anti-DYK agarose beads overnight at 4 °C. After washing five times, endogenous TTC3 or SMURF2 bound to SMURF2/Myc or TTC3/DYK, respectively, was detected by western blotting.

### Reverse transcription-polymerase chain reaction (RT-PCR)

Expression of *TTC3, SMURF2,* and *β-actin* was determined by RT-PCR. Total RNA from NHLFs, BEAS-2B cells, and mouse lungs were isolated by TRIzol reagent (Invitrogen). cDNA was synthesized with 1 μg of total RNA and murine leukemia virus reverse transcriptase (Intron, Seoul, Korea) according to the manufacturer’s instructions. PCR primer sequences were as follows: human *TTC3*, 5′-CCT GGC AAT GGA AGA AGC TC-3′ and 5′-AAT GAC CCT TTG GCC AAG TG-3′; human *ACTB* (β-actin), 5′-GAA AAT CTG GCA CCA CAC-3′ and 5′-GAT CTG GGT CAT CTT CTC-3′; mouse *Ttc3*, 5′-CAT GAC TGG AGC AGT CCT CA-3′ and 5′-GAC TGG CTT GAC CGA GTA GC-3′; mouse *Smurf2*, 5′- CATCGAAGCTCAGTTCCTGG-3′ and 5′-CGACATTGCTGTCTGGGGTA-3′; mouse *Actb*, 5′-GCC AAC CGT GAA AAG ATG AC-3′ and 5′-ATG AGG TAG TCT GTC AGG TC-3′. PCR products were separated in 1% agarose gels and analyzed using a Gel-Doc system (BioRad, Hercules, CA, USA), and image densities were quantified with ImageJ (https://imagej.net).

### Bleomycin-induced pulmonary fibrosis in mice

The animal experimental protocol in this study was reviewed and approved by the Institutional Animal Care and Use Committee of Sungkyunkwan University School of Medicine. After 8-week-old C57BL/6 male mice (Orient, Sungnam, Korea) were anesthetized with rumpun (10 mg/kg) and zoletil (25 mg/kg), their tracheas were surgically exposed, and bleomycin was injected with a 30 gauge-needle at a dose of 2 mg/kg. After 3 weeks of bleomycin administration, lungs were harvested and frozen in liquid nitrogen.

### Statistical analysis

Data from cell and mouse experiments are expressed as means ± SD and statistically analyzed by using SigmaPlot (Systat Software, San Jose, CA, USA), as described in the Supplemental Materials and Methods.

## Results

### TTC3 positively regulates TGF-β-induced EMT and myofibroblast differentiation in human bronchial epithelial cells and lung fibroblasts, respectively

**Fig. 1:**
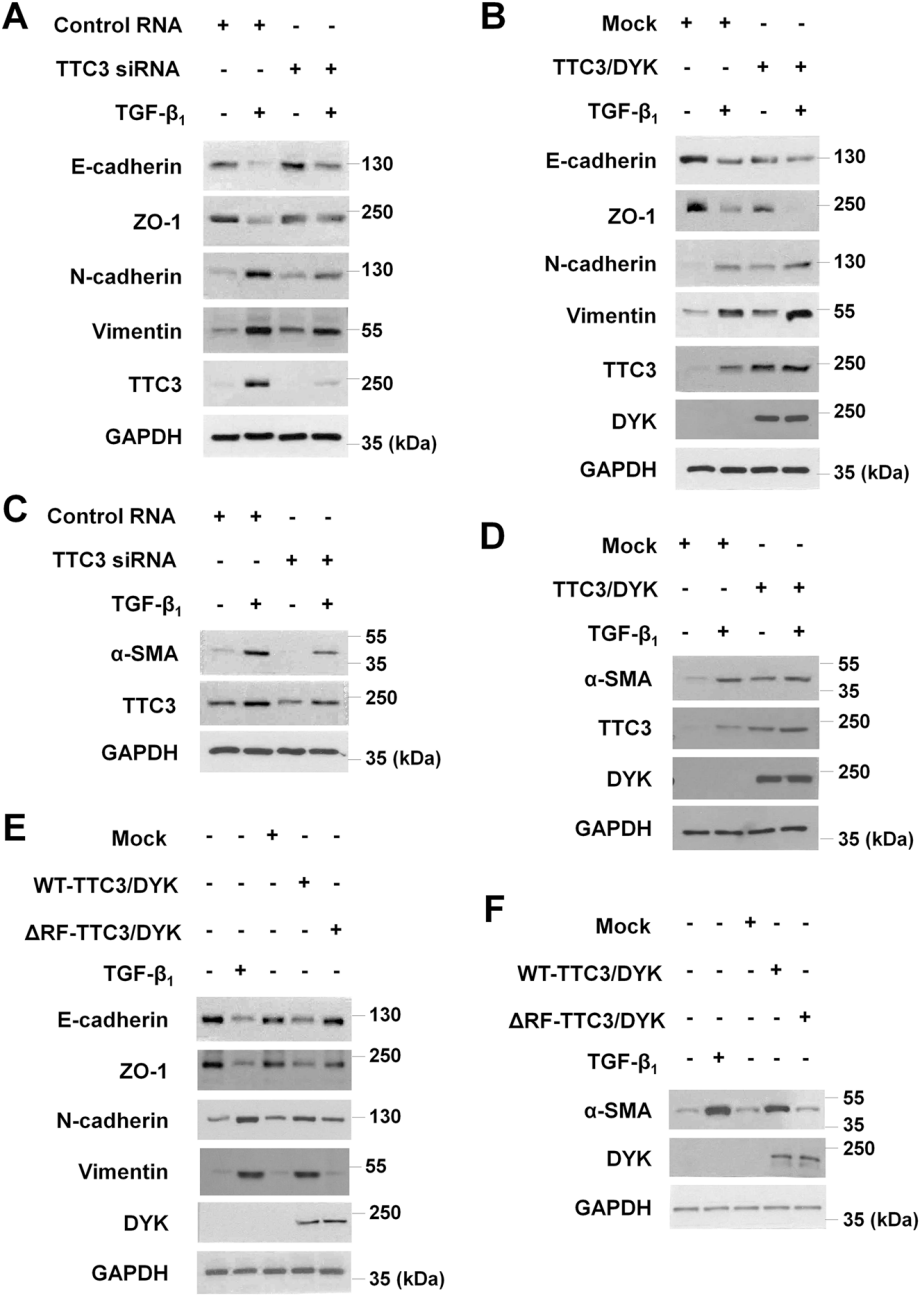
Tetratricopeptide repeat domain 3 (TTC3) positively regulates transforming growth factor-β_1_ (TGF-β_1_)-induced epithelial−mesenchymal transition (EMT) and myofibroblast differentiation in human bronchial epithelial cells (BEAS-2B) and normal human lung fibroblasts (NHLFs), respectively. Effects of TTC3 knockdown (**a**) and TTC3 overexpression (**b**) upon TGF-β_1_-induced EMT in BEAS-2B cells. One day after the transfection of TTC3 siRNA (**a**) and TTC3/DYK plasmid (**b**), BEAS-2B cells were treated with 10 ng/ml TGF-β_1_ for 1 d in the absence of serum. Effects of TTC3 knockdown (**c**) and TTC3 overexpression (**d**) upon TGF-β_1_-induced myofibroblast differentiation in NHLFs. TTC3 knockdown (**c**) and overexpression (**d**) and TGF-β_1_ treatment in NHLFs were performed as in BEAS-2B cells. Requirement of the TTC3 RING domain in the induction of EMT (**e**) and myofibroblast differentiation (**f**). BEAS-2B cells (**e**) and NHLFs (**f**) were transfected with mock, wild-type TTC3 (WT-TTC3/DYK), and TTC3 mutant (RING domain-deleted; 1956Cys to 1997Gln, ΔRF-TTC3/DYK) plasmids, and harvested 2 d after transfection. For comparison, BEAS-2B cells and NHLFs were treated with 10 ng/ml TGF-β_1_ for 1 d

Given that (1) TTC3 induced by cigarette smoke extract caused cell death, possibly through the ubiquitylation and degradation of Akt^17^, (2) cigarette smoking is associated with idiopathic pulmonary fibrosis (IPF)^22–24^, (3) Akt inhibition ameliorated pulmonary fibrosis in bleomycin-treated mice^25^, and (4) Akt is one of the noncanonical signaling arms of TGF- β^26^ that is responsible in fibrotic changes in IPF^27^, we hypothesized that TTC3 might affect TGF-β-mediated EMT and myofibroblast differentiation, characteristic features of fibrotic diseases including IPF^27^. In BEAS-2B cells (a human bronchial epithelial cell line), TGF-β_1_ decreased E-cadherin and ZO-1 (epithelial markers), while increasing N-cadherin and vimentin (mesenchymal markers), together with TTC3 induction (Fig. 1a and Supplemental Figs. 1a and 2). TTC3 siRNA targeting the coding region of *TTC3* significantly suppressed the TGF-β_1_-induced decrease in E-cadherin and ZO-1 and attenuated the TGF-β_1_-induced increase in N-cadherin and vimentin (Fig. 1a and Supplemental Fig. 1a). The suppressive effects of TTC3 siRNA were dose-dependent (Supplemental Fig. 3a), and another TTC3 siRNA targeting the 3′-UTR of *TTC3* also similarly suppressed TGF-β_1_-induced changes in E-cadherin, ZO-1, N-cadherin, and vimentin (Supplemental Fig. 4). Moreover, TTC3 overexpression amplified the TGF-β_1_-induced decrease in E-cadherin and ZO-1 and increase in N-cadherin and vimentin in BEAS-2B cells (Fig. 1b and Supplemental Fig. 1b). Interestingly, TTC3 overexpression alone caused EMT in a dose-dependentmanner (Fig. 1b and Supplemental Figs. 1b and 5).

Similarly, TTC3 also positively regulated TGF-β_1_-induced myofibroblast differentiation in NHLFs. TGF-β_1_ induced TTC3 and α-SMA, whereas TTC3 knockdown inhibited the TGF-β_1_-induced increase in α-SMA (Fig. 1c and Supplemental Figs. 1c and 2). TTC3 siRNA attenuated the TGF-β_1_-induced increase in α-SMA in a dose-dependent manner (Supplemental Fig. 3b), and TTC3 siRNA targeting the 3′-UTR of *TTC3* also suppressed the TGF-β_1_-induced increase in α-SMA (Supplemental Fig. 4b). Similar to the findings observed in BEAS-2B cells, TTC3 overexpression in the absence of TGF-β_1_ increased α-SMA (Fig. 1d and Supplemental Figs. 1d and 5), and potentiated TGF-β_1_-induced increase in α-SMA (Fig. 1d). In addition, TTC3 siRNA significantly inhibited both TGF-β_1_- or TTC3-induced migration of BEAS-2B cells (Supplemental Fig. 6). SB431542, an inhibitor of TGF-β receptor (TGFR) kinase, inhibited TGF-β_1_-induced EMT and myofibroblast differentiation, but not in TTC3-overexpressing cells (Supplemental Fig. 7). Taken together, these results imply that TTC3, downstream of TGFR, may positively regulate TGF-β_1_-induced EMT and myofibroblast differentiation.

Next, we assessed the requirement of the ubiquitin E3 ligase activity of TTC3 for the induction of EMT and myofibroblast differentiation. While overexpression of the wild-type TTC3 induced changes in E-cadherin, ZO-1, N-cadherin, and vimentin similar to TGF-β_1_ treatment, overexpression of the RING domain-deleted ΔRF-TTC3 did not change the levels of the EMT markers and α-SMA (Fig. 1e, f and Supplemental Fig. 1e, f).

### TTC3 positively regulates TGF-β signaling and induces proteasomal degradation of SMURF2

**Fig. 2:**
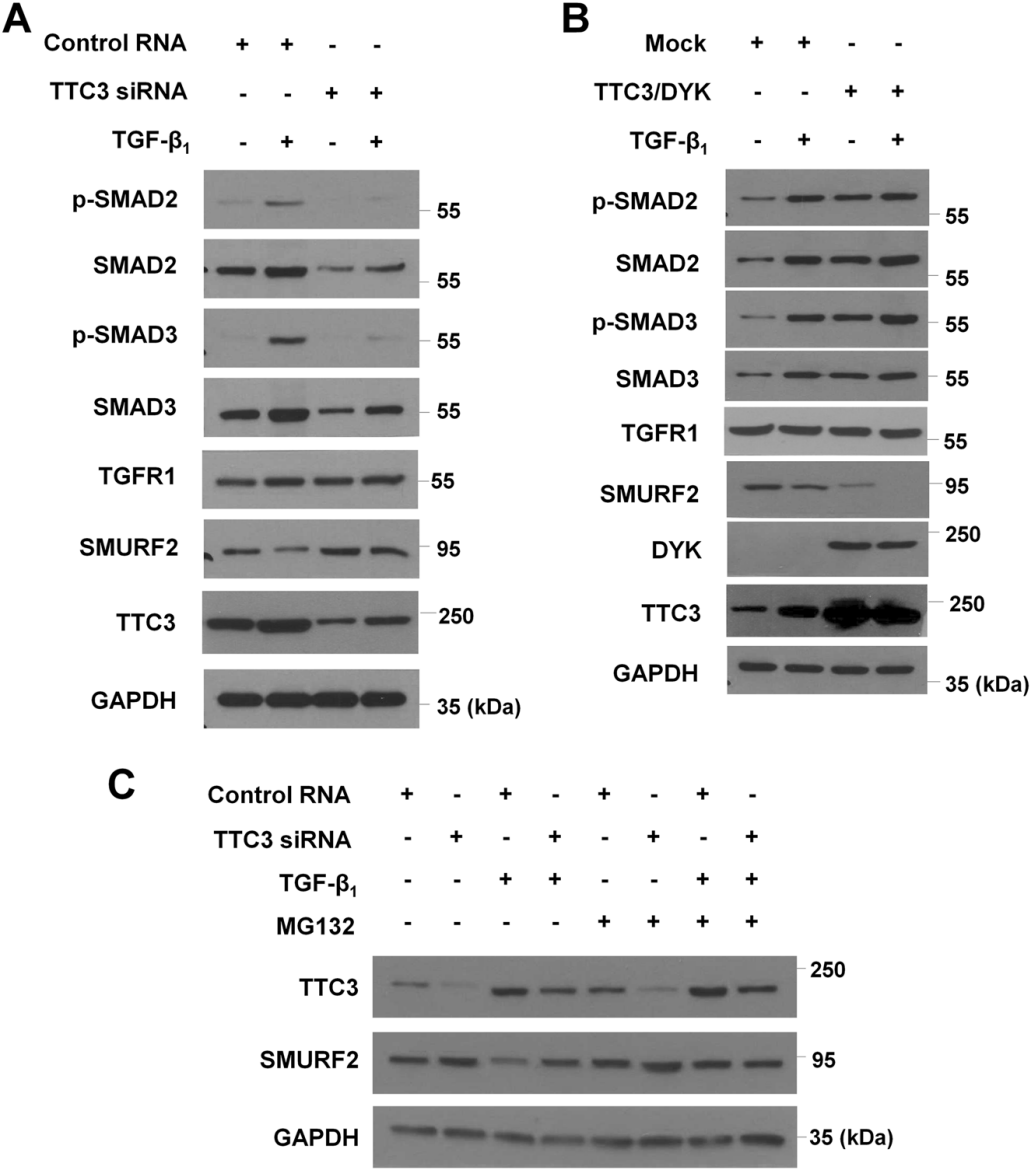
TTC3 positively regulates TGF-β signaling and TGF-β_1_ reduces SMURF2 in TTC3- and proteasome-dependent manners. Effects of TTC3 knockdown (**a**) and TTC3 overexpression (**b**) on TGF-β signaling. BEAS-2B cells were transfected with TTC3 siRNA (**a**) and TTC3/DYK plasmids (**b**) and treated with 10 ng/ml TGF-β_1_ for 1 d. **c** TTC3-mediated proteasomal degradation of SMURF2 in TGF-β_1_-treated BEAS-2B cells. After transfection of TTC3 siRNA, BEAS-2B cells were treated with for 10 ng/ml TGF-β_1_ for 1 d. MG132 (5 μM) was added 12 h before harvesting the cells

The above findings were contradictory to our initial expectation that TTC3 might suppress TGF-β_1_-induced EMT and myofibroblast differentiation through Akt ubiquitylation and degradation. Hence, we investigated whether the effects of TTC3 might be mediated independently of Akt ubiquitylation/degradation. In both BEAS-2B cells and NHLFs, TGF-β_1_ significantly activated Akt phosphorylation, while neither knockdown nor overexpression of TTC3 affected p-Akt and Akt levels (Supplemental Fig. 8a). Furthermore, overexpression of myristoylated Akt (Myr-Akt) did not inhibit the suppressive effect of TTC3 knockdown upon TGF-β_1_-induced EMT and myofibroblast differentiation (Supplemental Fig. 8b). As expected, phosphorylation of Akt substrates like glycogen synthase kinase 3α/β (GSK3α/β) were increased by Myr-Akt overexpression. In contrast, Akt1/2 knockdown inhibited TGF-β_1_-induced EMT and myofibroblast differentiation, similar to TTC3 knockdown (except ZO-1, Supplemental Fig. 8c). These data imply that TTC3 may act independently of Akt, while endogenous levels of Akt may be required in TGF-β_1_-induced EMT and myofibroblast differentiation. Next, to reveal a target of TTC3, we addressed whether TTC3 might enhance the canonical pathway of TGF-β signaling. In BEAS-2B cells, TTC3 siRNA suppressed the increase of both total and phosphorylated SMAD2 and SMAD3 under TGF-β_1_-stimulated conditions (Fig. 2a and Supplemental Fig. 9a). Similar to what was observed during EMT and myofibroblast differentiation in Fig. 1b, TTC3 overexpression itself elevated p-SMAD2, p-SMAD3, and SMAD3 in the absence of TGF-β_1_ treatment (Fig. 2b and Supplemental Fig. 9b) without further augmentation of increase in p-SMAD2, SMAD2, and p-SMAD3 except SMAD3 in comparison with TGF-β_1_-stimulation alone (Fig. 2b and Supplemental Fig. 9b). In contrast to SMAD2/3, TGF-β receptor 1 (TGFR1) levels were not changed by TGF-β_1_ treatment, TTC3 knockdown, or TTC3 overexpression. Considering the previous reports indicating that SMURF2 acts as a negative regulator of TGF-β signaling by modifying its related proteins including SMAD2 and SMAD3^11–14^, we addressed whether TTC3 might cause SMURF2 reduction. TTC3 knockdown suppressed TGFβ_1_-induced SMURF2 reduction (Fig. 2a and Supplemental Fig. 9a), while TTC3 overexpression by itself not only reduced the SMURF2 level, but also augmented TGF-β_1_-induced SMURF2 reduction (Fig. 2b and Supplemental Fig. 9b). MG132 treatment inhibited TGF-β_1_-induced SMURF2 reduction similar to that observed by TTC3 knockdown, even though MG132 did not inhibit TGF-β_1_-mediated TTC3 induction (Fig. 2c and Supplemental Fig. 9c). In time-chase experiments, MG132 inhibited SMURF2 reduction in TTC3-overexpressing BEAS-2B cells, while cycloheximide did not (Supplemental Fig. 10). Taken together, we hypothesized that TGF-β_1_-induced TTC3 might lead to the ubiquitylation and proteasomal degradation of SMURF2.

### TTC3 ubiquitylates SMURF2 in a Lys48-linked manner

**Fig. 3:**
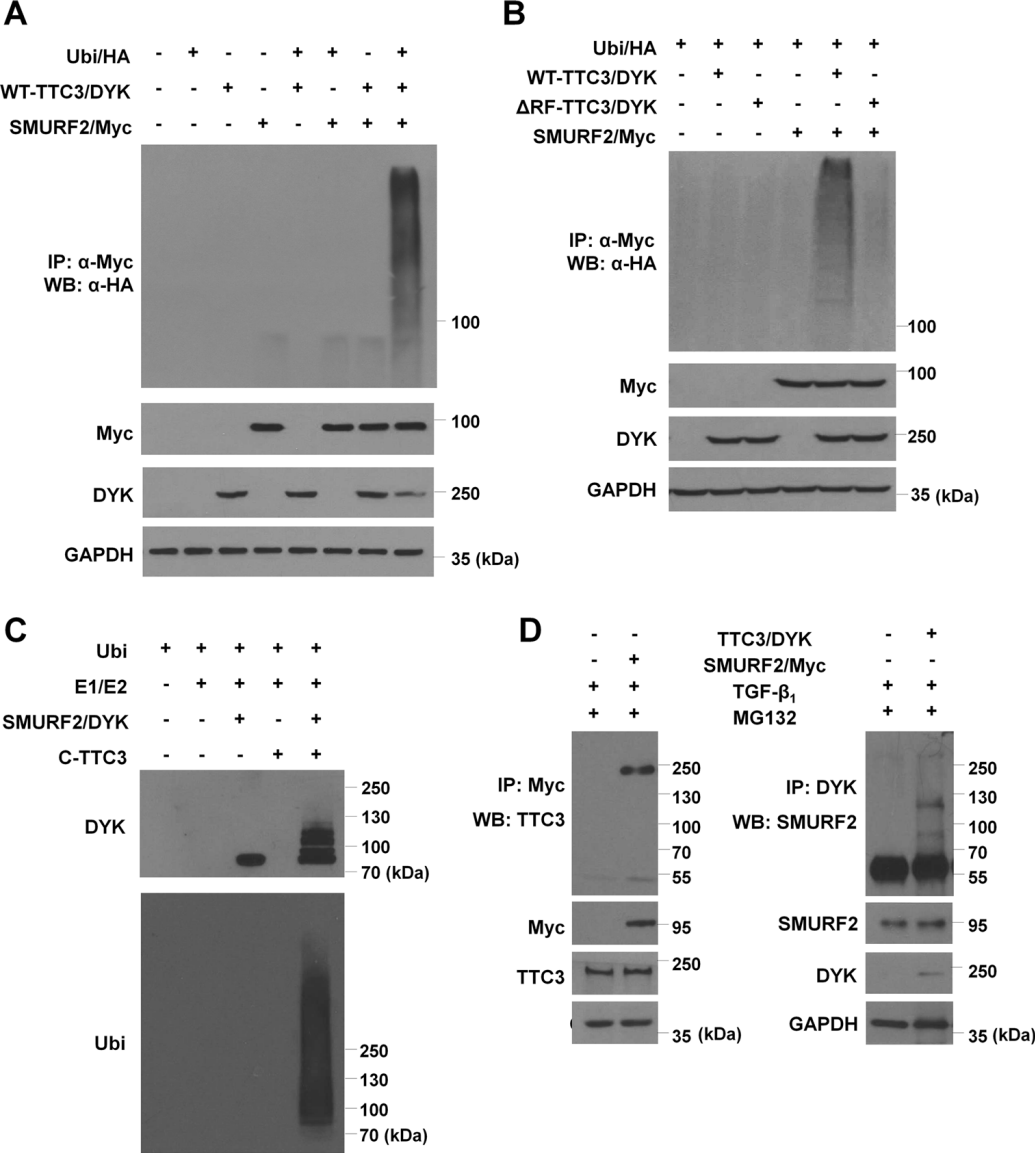
TTC3-mediated SMURF2 ubiquitylation. **a** TTC3-mediated SMURF2 ubiquitylation in HEK293 cells. HEK293 cells were transfected with TTC3/DYK plasmids on day 1 and transfected with SMURF2/Myc and Ubi/HA plasmids on day 2. After an overnight incubation, 5 μM MG132 was added and the cells were harvested on day 4. SMURF2/Myc was immunoprecipitated with anti-Myc antibody-conjugated agarose beads and ubiquitylated SMURF2 was detected with an anti-HA antibody. **b** Requirement of the RING domain of TTC3 for SMURF2 ubiquitylation in HEK293 cells. HEK293 cells were transfected with the wild-type TTC3 (WT-TTC3/DYK) and RING domain-deleted mutant TTC3 (ΔRF-TTC3/DDK), and the cell ubiquitylation assay was done as in (**a**). **c** TTC3-mediated SMURF2 ubiquitylation in vitro. Recombinant human SMURF2/DYK was incubated with 6×-His recombinant C-terminal wild-type TTC3 (WT-C-TTC3; Ile1540 to Arg2025) with human E1 (UBE1) and E2 (UBCE2E3) for 4 h at 30 °C. Ubiquitylated SMURF2/DYK was detected by western blotting using an anti-ubiquitin antibody. **d** Interaction between SMURF2 and TTC3 in BEAS-2B cells. BEAS-2B cells were transfected with either SMURF2/Myc or TTC3/DYK, and treated with 10 ng/ml TGF-β_1_ for 1 d. After cell lysis, SMURF2 and TTC3 were immunoprecipitated with anti-Myc or anti-DYK antibodies, respectively. Bound TTC3 and SMURF2 were detected by western blotting

To test our hypothesis, we performed in cell and in vitro ubiquitylation and coimmunoprecipitation assays. In HEK293 cells transfected with the wild-type TTC3 (WT-TTC3/DYK), SMURF2 (SMURF2/Myc), and ubiquitin (Ubi/HA) plasmids, SMURF2 ubiquitylation was clearly observed as compared to the other samples missing one or more plasmids, while the mutant TTC3 (ΔRF-TTC3/DYK) did not cause SMURF2 ubiquitylation (Fig. 3a, b). In the in vitro ubiquitylation assay, the recombinant wild-type C-terminal TTC3 (C-TTC3) caused SMURF2 ubiquitylation (Fig. 3c). In BEAS-2B cells stimulated with TGF-β_1_, endogenous TTC3 and SMURF2 were coimmunoprecipitated with SMURF2/Myc and TTC3/DYK, respectively (Fig. 3d). Consistently, we observed that endogenous TTC3 bound to endogenous SMURF2 in BEAS-2B cells treated with TGF-β_1_ (Supplemental Fig. 11). TTC3-mediated SMURF2 ubiquitylation occurred mainly in a Lys48-linked manner, as SMURF2 ubiquitylation was observed when WT-Ubi (the wild-type ubiquitin) and K48-Ubi (all Lys replaced with Arg except Lys48) were expressed (Supplemental Fig. 12). As SMURF2 is known to be autoubiquitylated^28^, we assessed whether TTC3 might affect SMURF2 autoubiquitylation by comparing ubiquitylation among the wild-type (WT), catalytically inactive (C716A), and active (FF29/30A) SMURF2. In the absence of TTC3, FF29/30A-SMURF2 was autoubiquitylated as expected, whereas ubiquitylation of WT-SMURF was marginal and ubiquitylation of C716A-SMURF was not seen (Supplemental Fig. 13). In the presence of TTC3, ubiquitylation was enhanced in all forms of SMURF2. Compared with WT- and FF29/30A-SMURF2, C716A-SMURF2 was less ubiquitylated by TTC3 and less bound to TTC3. Taken together, TTC3 may directly ubiquitylate SMURF2, and the catalytic domain in SMURF2 may be important in the interaction between TTC3 and SMURF2, thus enabling TTC3-mediated SMURF2 ubiquitylation.

### SMURF2 counteracts TTC3 in EMT and myofibroblast differentiation

**Fig. 4:**
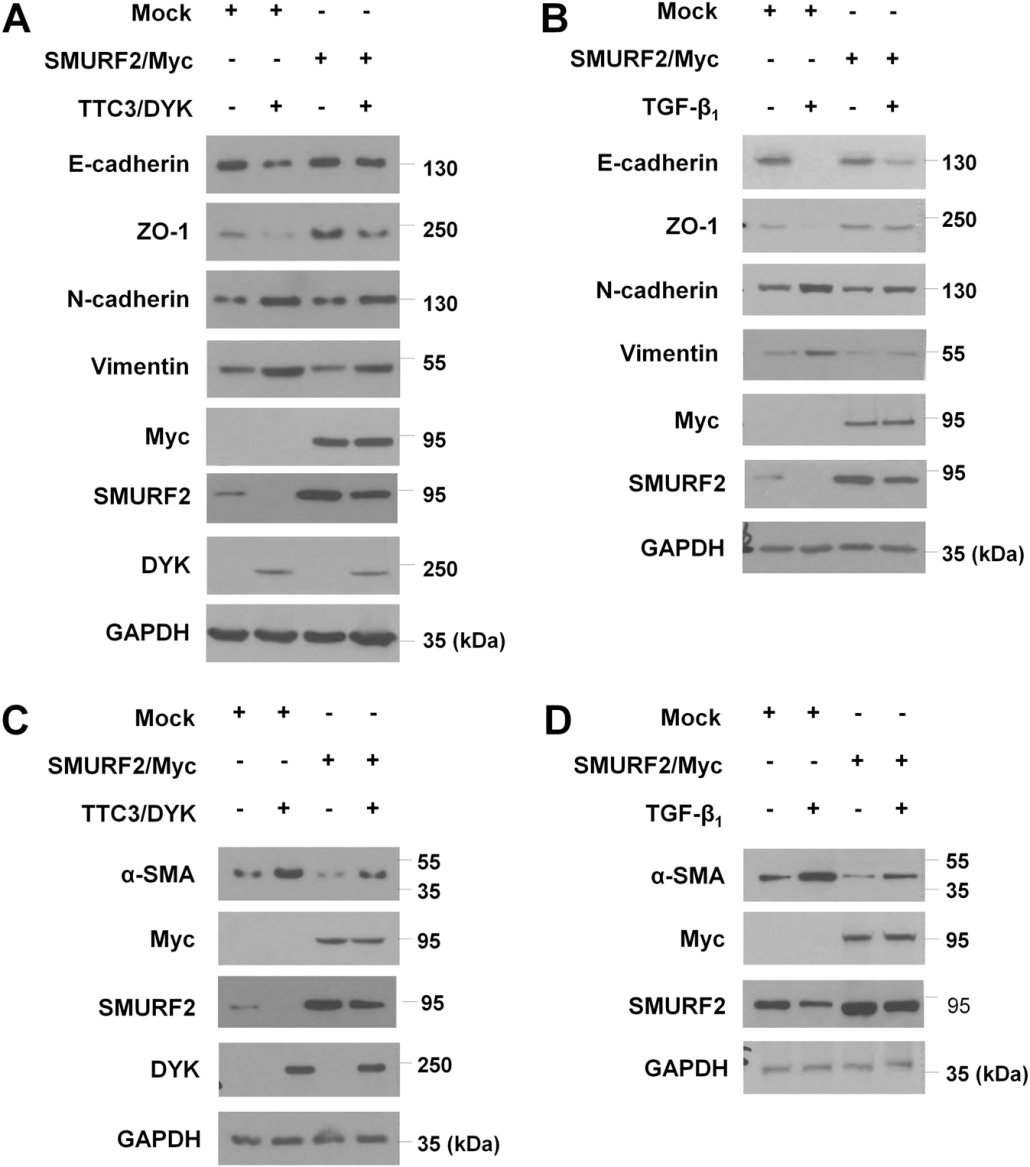
SMURF2 overexpression inhibits EMT and myofibroblast differentiation induced by TTC3 overexpression and TGF-β_1_ treatment. To assess the effects of SMURF2 upon TTC3 overexpression, BEAS-2B cells (**a**) and NHLFs (**c**) were transfected with TTC3/DYK plasmid and 12 h later transfected with SMURF2/Myc plasmid, and both cells were harvested 2 d later. To assess the effects of SMURF2 upon treatment with TGF-β_1_, BEAS-2B cells (**b**) and NHLFs (**d**) were transfected with SMURF2/Myc plasmid, and the serum-deprived cells were treated with 10 ng/ml TGF-β_1_ for 1 d

To confirm the positive regulatory role of TTC3 in EMT and myofibroblast differentiation through SMURF2 reduction, we assessed whether SMURF2 overexpression could counteract TTC3 overexpression and TGF-β_1_ stimulation. In BEAS-2B cells and NHLFs, SMURF2 overexpression inhibited the TTC3- and TGF-β_1_-induced EMT and myofibroblast differentiation, respectively (Fig. 4 and Supplemental Fig. 14). The suppressive effects of SMURF2 upon in TGF-β_1_- and TTC3-induced EMT and myofibroblast differentiation was dose-dependent (Supplemental Fig. 15). Moreover, SMURF2 siRNA suppressed the inhibitory effects of TTC3 siRNA upon TGF-β_1_-induced EMT, myofibroblast differentiation, and increase in p-SMAD2/3 and SMAD2/3 (Supplemental Fig. 16). These data verify the idea that SMURF2 may be the downstream target of TTC3 in the context of TGF-β_1_-induced EMT and myofibroblast differentiation.

**Fig. 5:**
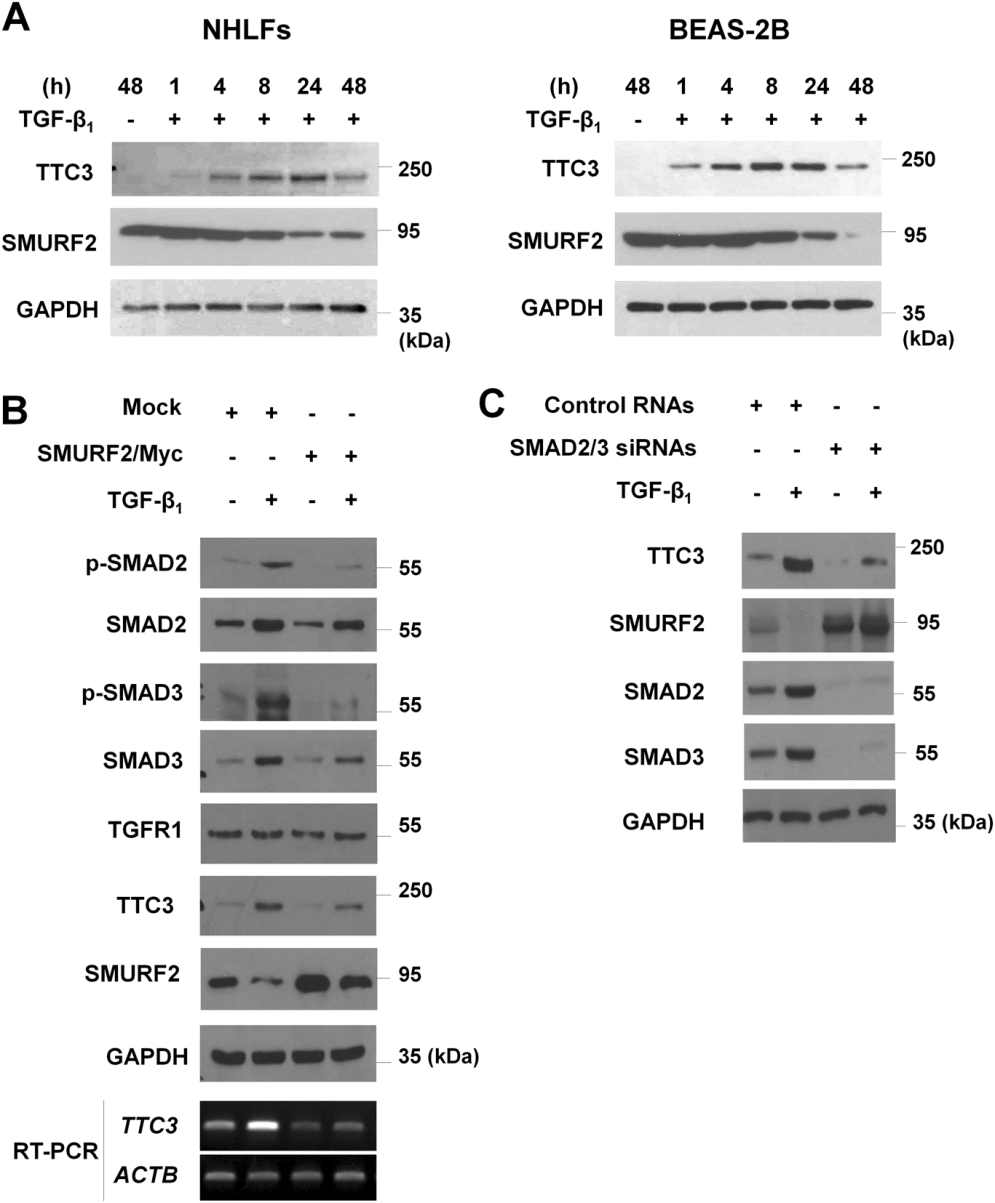
TGF-β_1_-induced TTC3 expression through SMAD2/3. **a** A time-course of TGF-β_1_-induced TTC3 expression in BEAS-2B cells and NHLFs. BEAS-2B cells and NHLFs were treated with 10 ng/ml TGF-β_1_ and harvested at the indicated time points. **b** Inhibition of TGF-β_1_-induced elevation of total and phosphorylated forms of SMAD2 and SMAD3 in BEAS-2B cells by SMURF2 overexpression. After transfection of SMURF2/Myc, BEAS-2B cells were treated with 10 ng/ml TGF-β_1_ for 1 d. **c** SMAD2/3-mediated TTC3 induction in TGF-β_1_-treated BEAS-2B cells. BEAS-2B cells were transfected with SMAD2 and SMAD3 siRNAs and treated with 10 ng/ml TGF-β_1_ for 1 d

Interestingly, TGF-β_1_-mediated TTC3 induction was also suppressed by SMURF2 overexpression. While TGF-β_1_ induced TTC3 expression and reduced SMURF2 levels in a time-dependent manner (Fig. 5a and Supplemental Fig. 17a), SMURF2 overexpression not only reduced TGF-β_1_-induced elevation of total and phosphorylated SMAD2 and SMAD3, but also suppressed TGF-β_1_-induced elevation of TTC3 mRNA and protein (Fig. 5b and Supplemental Fig. 17b). The inhibitory effects of SMURF2 overexpression upon TGF-β_1_-induced TTC3 expression were dose-dependent (Supplemental Fig. 15). Moreover, knockdown of SMAD2 and SMAD3 attenuated TGF-β_1_-induced TTC3 expression, while inhibiting TGF-β_1_-induced SMURF2 reduction (Fig. 5c and Supplemental Fig. 17c). However, SMURF2 overexpression did not affect levels of p-Akt, Akt, and TGFR1 levels in the absence and presence of TGF-β_1_ (Supplemental Fig. 8d and 17b). Taken together, these data imply that TGF-β_1_-induced TTC3 may positively regulate TGF-β signaling in a feedforward mechanism through SMURF2 ubiquitylation/degradation and the subsequent elevation of SMAD2 and SMAD3, which in turn induces TTC3 expression.

### Changes in *Ttc3* mRNA and Smurf2 protein levels in the lungs of bleomycin-treated mice

**Fig. 6:**
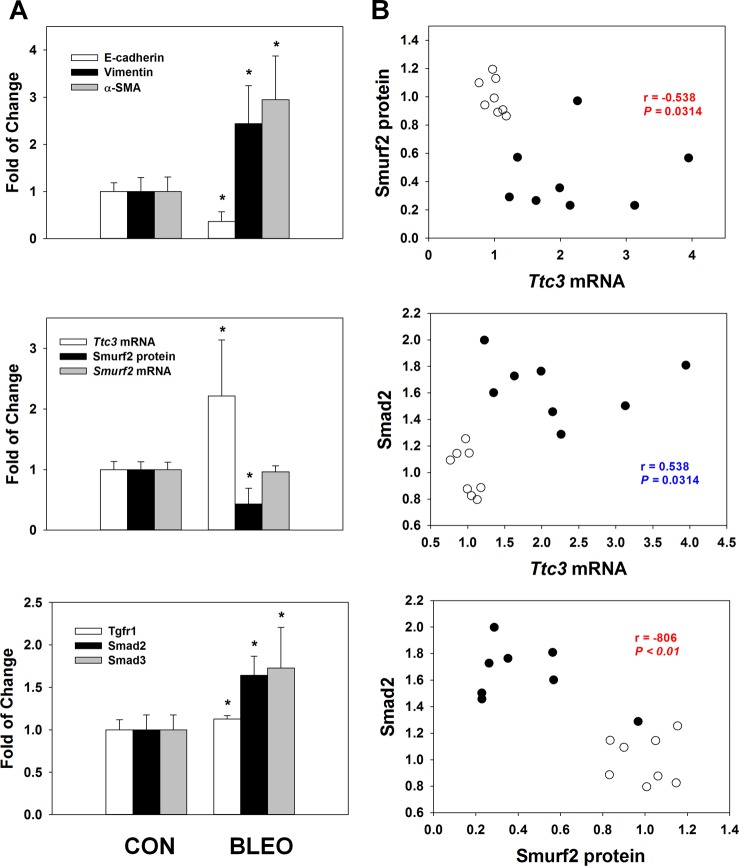
Changes in the levels of *Ttc3* mRNA, *Smurf2* mRNA, proteins Smurf2, Smad2, Smad3, Tgfr1, E-cadherin, vimentin, and α-SMA in mouse lungs. Bleomycin (2 mg/kg) was administered to 8-week-old male C57BL/6 mice through their trachea under anesthesia. After 3 weeks of bleomycin administration, lungs were harvested. **a** Levels of *Ttc3* mRNA, Smurf2 mRNA, proteins Smurf2, Smad2, Smad3, Tgfr1, E-cadherin, vimentin, and α-SMA. Data are expressed as means ± SD. Asterisks indicate statistically significant difference between the CON and BLEO groups (*N* = 8 in each group, *P* *<* 0.01). **b** Correlation analyses. All the data from both CON (blank circle) and BLEO (black circle) groups were collected together for correlation analysis

As EMT and myofibroblast differentiation have been suggested to play a role in fibrotic diseases including pulmonary fibrosis^7,29^, we investigated the changes in Ttc3 and Smurf2 in the lungs of bleomycin-administered mice, an animal model of pulmonary fibrosis^30^. Three weeks after bleomycin administration, E-cadherin levels were significantly lower in the bleomycin-treated group (BLEO) than in the control group (CON), and the levels of vimentin and α-SMA were significantly higher than those of the CON group (Fig. 6a). Using fluorescent immunohistochemistry, decreased E-cadherin and increased vimentin and α-SMA in alveolar epithelium and fibrotic area were observed in the BLEO group, compared with the CON group (Supplemental Fig. 18). *Ttc3* mRNA and Smurf2 protein levels in the BLEO group were significantly higher than those in the CON group, while there was no significant difference in *Smurf2* mRNA levels between the two groups (Fig. 6a). Smad2 and Smad3 levels were significantly higher in the BLEO group than those in the CON group. Increase in Tgfr1 levels in the BLEO group was marginal, but significant, compared with that in the CON group. In correlation analyses, *Ttc3* mRNA levels were negatively correlated with Smurf2 protein levels and positively correlated with Smad2 levels, while Smurf2 protein levels were negatively correlated with Smad2 and Tgfr1 (Fig. 6b and Supplemental Table 2). In addition, *Ttc3* mRNA levels were negatively correlated with E-cadherin, while Smurf2 protein levels were positively correlated with E-cadherin and negatively correlated with vimentin and α-SMA (Supplemental Fig. 19 and Supplemental Table 3). Finally, bleomycin treatment in BEAS-2B cells and NHLFs induced EMT and myofibroblast differentiation together with TTC3 induction and TTC3 knockdown suppressed the bleomycin-induced EMT and myofibroblast differentiation (Supplemental Fig. 20).

## Discussion

Ubiquitylation is one of regulatory mechanisms of TGF-β signaling, and SMURF2 plays an important role as it causes the ubiquitylation of components in the TGF-β signaling pathway. SMURF2 induces SMAD2 ubiquitylation and proteasome-dependent degradation, and consequently reduces the transcriptional activity of SMAD2 ^11,12^. SMURF2, which is mainly localized in the nucleus, binds to SMAD7, translocates out of the nucleus^31^, and induces turnover of TGF-β receptors^10,32^. In addition, SMURF2 causes multiple monoubiquitylation of SMAD3 and inhibits the formation of active SMAD3 complexes, instead of polyubiquitylation and proteasomal degradation of SMAD3^14^. By contrast, SMURF2 overexpression had little effect on the basal or TGF-β-stimulated p-Smad2, Smad2, or TGFR1 levels, while there was a noticeable decrease in the level of p-SMAD3 and ubiquitylation of p-SMAD3 in sternal chondrocytes obtained from *Smurf2* transgenic mice^13^. This discrepancy in SMAD3 ubiquitylation may originate from the difference in cell types or experimental settings. Alternatively, SMURF2 may play an auxiliary role in the degradative ubiquitylation caused by other SMAD3 ubiquitin E3 ligases such as CHIP^33^, Arkadia^34^, or ROC-1 ^35^.

The activity of SMURF2 is known to be positively regulated by several mechanisms. SMAD7 antagonizes the autoinhibitory interaction between the C2 and HECT domains of SMURF2 by binding with the HECT-binding domain of SMAD7. This leads to SMURF2 activation^28^, and SMAD7 stimulates SMURF2 activity by recruiting the E2 enzyme, UbcH7, to the HECT domain of SMURF2^36^. Similarly, PIN1 increased the interaction between SMURF2 and SMAD2/3 and induced SMAD2/3 ubiquitylation^37^. In addition, SUMOylation of SMURF2 enhances SMURF2-mediated TGFR degradation, thereby suppressing the TGF-β/SMAD pathway and EMT of mammary epithelial cells^38^. Similarly, noncovalent binding of NEDD8 within the HECT domain of SMURF2 is important in decreasing SMAD3 and p-SMAD3 and suppressing TGF-β signaling activity^39^.

In contrast to the enhancing mechanisms of SMURF2 activity, a few negative regulatory mechanisms have been reported. The SMAD7 binding to SMURF2 results not only in the activation of E3 ligase activity of SMURF2, but also induction of its autoubiquitylation^28^. RNF11 also acts as a negative regulator of SMURF2 by competing with SMAD7 for SMURF2 binding in a mutually exclusive manner, consequently antagonizing SMURF2 and positively regulating TGF-β signaling^40^. Upon TGF-β stimulation, TRAF4 is recruited to TGFR1 and antagonizes SMURF2 through polyubiquitylation/degradation^41^. TRB3 interacts with SMURF2 and promotes its ubiquitylation/degradation, causes the accumulation of SMAD3 in the nucleus^42^. TRB3 is induced in a TGF-β/SMAD3-dependent manner, and thus is a feedforward positive regulator of TGF-β signaling, similar to TTC3. However, it remains unknown which ubiquitin E3 ligase cooperates with TRB3 for the SMURF2 ubiquitylation.

As TGF-β plays a critical role in EMT, researchers have suggested that SMURF2 may play an important regulatory role in EMT. Zhang et al. demonstrated that TRAF4, a SMURF2 ubiquitin E3 ligase, enhanced TGF-β-induced EMT, cell invasion, and cell migration^41^. They also reported that metastasis-free survival is higher in the breast cancer patient group with lower TRAF4 expression compared to the breast cancer patient group with higher TRAF4 expression. Moreover, OTUD1 suppresses TGF-β signaling and EMT in MCF10A cells expressing RAS and metastasis of breast cancer cells in mice, respectively, presumably through deubiquitylating SMAD7 thus enabling SMURF2 binding and subsequent TGFR turnover^43^. Consistently, SMURF2 suppressed TGF-β-induced EMT in three-dimensional culture of mammary NMuMG epithelial cells^38^. In contrast, SMURF2 overexpression enhanced TGF-β-induced N-cadherin expression and cell migration/invasion in MCF10Ca1a cells (metastatic breast cancer cells)^44^. The discrepancy may originate from the difference in the cells used in these studies. Alternatively, there may be other SMURF2 targets that may indirectly influence TGF-β pathways and EMT, such as β-TrCP^45^ and axin^46^.

Similar to the conflicting notions regarding the role of SMURF2 in EMT under different experimental settings, a limited number of studies have investigated the intriguing roles of SMURF2 in organ fibrosis. In unilateral ureteral obstruction (UUO), a model of progressive interstitial fibrosis of the kidney, the levels of SMURF2 were increased. However, SMAD7, SnoN, and Ski, which are negative regulators of TGF-β signaling and SMURF2 substrates, decreased^47,48^. The authors suggested that SMURF2-mediated ubiquitylation/degradation of SMAD7, SnoN, and Ski might potentiate TGF-β signaling, leading to renal fibrosis. In human renal proximal tubular epithelial cells (HKC, clone 8), TGF-β_1_ elevated SMURF2 expression, and SMURF2 induced proteasome-dependent degradation of SMAD2, SnoN, and Ski^49^. Even though TGF-β_1_ caused the degradation of both TGF-β signaling proteins and negative regulatory proteins, the net result of SMURF2 overexpression in TGF-β_1_-treated HKC-8 cells was enhanced TGF-β_1_-mediated transcriptional induction and EMT phenotypes. Hence, the authors suggested that dysregulation of SMURF2 could contribute to the pathogenesis of renal fibrosis. However, Liu et al. suggested that Arkadia might be responsible for the TGF-β-mediated reduction in SMDA7 rather than SMURF2 in HKCs^50^. Compared to UUO kidneys^47,48^ and human renal fibrotic kidneys^49^, scleroderma fibroblasts obtained from the affected areas exhibited increased SMAD7 levels and TGFR1 stability, while there was no significant difference in SMURF2 levels compared with normal fibroblasts from healthy volunteers^51^. Interestingly, overexpression of SMURF1/2 did not change TGFR1 levels under basal and TGF-β-stimulated conditions in scleroderma fibroblasts, while it reduced both basal and TGF-β stimulated levels of TGFR1 in normal skin fibroblasts. The authors suggested that impairment of TGFR turnover even with SMURF2-SMAD7-TGFR complex formation might contribute to enhanced TGF-β signaling. Compared with the above studies focusing on the SMURF2 targets within the TGF-β pathway, Cai et al. recently reported a novel mechanism of SMURF2 action in an animal model of liver fibrosis^52^. SMURF2 overexpression in the liver alleviated fibrotic changes including collagen deposition, myofibroblast differentiation, and connective tissue growth factor (CTGF) induction. As a mechanism, the investigators proposed that SMURF2-mediated ubiquitylation/degradation of phosphodiesterase 4B, and the subsequent cAMP-induced expression of miR-132 inhibits CTGF expression. To our knowledge, a pathophysiological role of SMURF2 in pulmonary fibrosis remains unknown, except for the data that SMURF2 expression was induced in both normal and IPF lung fibroblasts to a similar degree^53^, and that miR-424 that was induced by TGF-β_1_ decreased SMURF2 levels and positively regulated myofibroblast differentiation^54^.

TTC3 was first reported to act as an Akt ubiquitin E3 ligase^16^. However, both TTC3 knockdown and overexpression did not affect TGF-β_1_-induced Akt phosphorylation and overexpression of Myr-Akt did not suppress the inhibitory effects of TTC3 siRNA. By contrast, Akt knockdown inhibited TGF-β_1_-induced EMT and myofibroblast differentiation, similar to TTC3 knockdown. The results suggest that endogenous levels of Akt may allow TGF-β signaling to work in EMT and myofibroblast differentiation. For example, GSK-3β, which is negatively regulated by Akt, phosphorylates dual sites of Snail, which is a key transcriptional factor in EMT. This leads to the cytoplasmic localization of Snail and β-TrCP-mediated ubiquitylation/degradation^55^. Moreover, Akt phosphorylates Twist1, another key transcriptional factor in EMT, promoting TGF-β signaling and EMT^56^.

Here, we demonstrated for the first time that TTC3 mediates TGF-β_1_-induced EMT and myofibroblast differentiation, potentially through SMURF2 ubiquitylation/proteasomal degradation and subsequent inhibition of SMURF2-mediated suppression of SMAD2 and SMAD3, which in turn induces TTC3 expression (Supplemental Fig. 21). Moreover, we observed that *Ttc3* mRNA was induced, while Smurf2 protein levels were attenuated in the lungs of bleomycin-treated mice with significant correlation with TGF-β signaling molecules and EMT/myofibroblast differentiation-related molecules. With respect to the causal relationship between TTC3 and IPF, future investigation is needed to clarify whether TTC3 may be induced in IPF lungs and whether TTC3 inhibition is a viable therapeutic strategy. SMURF2 plays an important role in various physiological and pathological contexts: development, proliferation/apoptosis, senescence, tumorigenesis/tumor suppression, and metastasis^57^. Thus, TTC3-mediated SMURF2 ubiquitylation/degradation may provide novel insights into these research fields.

## Supplementary information

Supplemental document

Supplemental figures
