# Supplementary material for: TTC3 contributes to TGF-β1-induced epithelial−mesenchymal transition and myofibroblast differentiation, potentially through SMURF2 ubiquitylation and degradation
Source: Cell Death Dis. 2019 Jan 29;10(2):92. doi: 10.1038/s41419-019-1308-8 (PMC6351531; doi:10.1038/s41419-019-1308-8)
Supplement: Supplementary file 1 — Supplemental document [file 41419_2019_1308_MOESM1_ESM.docx]

**TTC3 contributes to TGF-β_1_-induced epithelial-mesenchymal transition and myofibroblast differentiation, possibly through SMURF2 ubiquitylation and degradation**

**June-Hyung Kim^1^, Sangwoo Ham^1^, Yunjong Lee^1^, Gee Young Suh^2, *^, Yun-Song Lee^1, *^**

1. Division of Pharmacology, Department of Molecular & Cellular Biology, Sungkyunkwan University School of Medicine, Samsung Biomedical Research Institute, Suwon, Republic of Korea
2. Department of Critical Care Medicine in Samsung Medical Center, Sungkyunkwan University School of Medicine, Seoul, Republic of Korea

**Supplemental Materials & Methods**

- SB 431542 and cycloheximide were purchased from Sigma (St. Louis, MO, USA). Protein A/G agarose beads were purchased from (Calbiochem, San Diego, CA, USA). Plasmids of the active SMURF2 (pCMV-Myc-SMURF2-FF29/30A, #24604) and catalytically inactive SMURF2 (pCMV5B-Flag-SMURF2-C716A, #11747) were gifts from Jeff Wrana (Addgene, Watertown, MA, USA)^1^. Both the active and inactive plasmids were subcloned into the plasmid with the same backbone of the wild-type SMURF2 plasmid. The sequences of the TTC3 siRNA targeting coding region^2^ and 3’-untranslated region (UTR) are 5’-AAGTATGCATGCAGTCTTGTA-3’ and 5’-TACAAGACTGCATGCATACTT-3’, respectively. SMAD2 siRNA (L-020067-00) and SMAD3 siRNA (L-003561-00)^3^ and SMURF2 siRNA (L-007194-00)^4^ were purchased from Dharmacon (Lafayette, CO, USA). Antibodies used in this study are in the following table.

**Methods**

**Cell migration assay**

BEAS-2B cells were seeded at a density of 12 000 cells per well of chamber slides (ibidi, Martinsried, Germany), and transfected with plasmid and siRNA. After overnight serum starvation, TGF-β_1_ was added, and barriers were removed. After 2 d, images of wound area were taken with an inverted microscope (Olympus, Tokyo, Japan), and analyzed using software (Adobe Photoshop, San Jose, CA, USA), similar to a previous report^5^. The wound area of the control cells was set at 100%.

**Co-immunoprecipitation assay**

Binding between endogenous TTC3 and SMURF2 in BEAS-2B cells was assessed by co-immunoprecipitation assay. BEAS-2B cells were treated with 10 ng/ml TGF-β_1_ for 1 d and 5 μM MG132 for 12 h before harvesting cells. Harvested cells were lysed in the Brij lysis buffer by freezing/thawing five times, and cell lysates were subject to centrifugation at 18 000 × *g* at 4 °C for 20 min. A total of 5.5 mg proteins in 800 μl binding buffer (lysis buffer of Cell signaling Technology (“CST buffer”):Brij lysis buffer = 2:1) was incubated with 20 μl of protein A/G beads for 1 h for preclearing. Precleared lysates were then incubated overnight with 20 μl of anti-SMURF2 antibody and 20 μl of protein A/G beads at 4 °C. After washing five times with the CST lysis buffer, endogenous TTC3 bound to SMURF was detected by western blot.

**Immunohistochemistry**

The immunohistochemistry protocol was adapted from previous reports with slight modification^6^. After three weeks of bleomycin administration, mice were anesthetized with intraperitoneal injections of ketamine and xylazine, and the heart and the lungs were removed *en bloc*. The right lung was inflated by intratracheal infusion of 4% paraformaldehyde in PBS at 20 cm H_2_O and embedded in paraffin. The left lung was frozen in liquid nitrogen for biochemical analysis. Lung sections of 4-μm thickness were deparaffinized and hydrated, and antigen retrieval was performed. The sections were incubated with a blocking solution (10% normal goat serum, Abcam; 0.1% Triton X-100) in TBS-T at room temperature for 30 min, and then incubated overnight with anti-SMURF2 (1:100), anti-E-cadherin (1:500), anti-vimentin (1:500), and anti-α-SMA (1:500) antibodies in the blocking solution at 4°C. The next day, the sections were washed with PBS containing 0.1% Triton X-100 and incubated with Alexa Fluor 586- and Alexa Fluor 488-conjugated secondary antibodies (1:500, Invitrogen, Carlsbad, CA, USA) in the blocking solution at room temperature for 1 h. After being washed, images were observed with a fluorescent microscope (Zeiss).

**Bleomycin-cell model**

After transfection of 200 pmols TTC3 siRNA or 10 μg SMURF2 plasmid per 10 cm culture dish, culture medium was replaced with fresh medium, and cells were exposed to 1 μg/ml bleomycin for one day, as described previously^7^.

**Statistical analysis**

Data obtained from western blotting and RT-PCR were normalized with GAPDH and β-Actin, respectively, and expressed as means ± SD. Multiple comparison of data from cell experiments was done by Holm-Sidak or Tukey’s methods, and data from mouse experiments were analyzed by one-tailed Student’s *t*-test or Mann-Whitney rank sum test. Correlation of data from mouse experiments was analyzed by the Pearson’s product moment correlation method.

**Supplemental Tables**

**Supplemental Table 1. Information of antibodies**

| **Name** | **Company** | **Cat#** | **Titer** |
| --- | --- | --- | --- |
| E-cadherin (24E10) | Cell Signaling | 3195 | 1:1000 in WB |
| ZO-1 (D7D12) | Cell Signaling | 8193 |  |
| N-cadherin (D4R1H) XP® | Cell signaling | 13116 |  |
| vimentin (n21H3) XP® | Cell Signaling | 5741 |  |
| SMAD2 (D43B4) XP® | Cell Signaling | 5339 |  |
| SMAD3 (C67H9) | Cell Signaling | 9523 |  |
| phospho-SMAD2 (S465/467) (138D4) | Cell Signaling | 3108 |  |
| phospho-SMAD3 (S423/425) (C25A9) | Cell Signaling | 9520 |  |
| TGFR1 | Abcam | ab31013 |  |
| SMURF2 (D8B8) | Cell Signaling | 12024 |  |
| Akt | Cell Signaling | 9272 |  |
| p-Akt | Cell Signaling | 9271 |  |
| GSK-3α/β(D75D3) | Cell Signaling | 5676 |  |
| p-GSK-3β(Ser9) | Cell Signaling | 9336 |  |
| HRP-conjugated anti-Rabbit IgG | Cell Signaling | 7074 | 1:5000 in WB |
| HRP-conjugated anti-Mouse IgG | Cell Signaling | 7076 |  |
| TTC3 | SIGMA | HPA016810 | 1:200 in WB |
| HA | SIGMA | H9658 | 1:1000 in WB |
| DYK (FLAG, M2) | SIGMA | F3165 |  |
| Myc | SIGMA | M4439 |  |
| GAPDH | AbFrontier | LF-PA0018 |  |
| α-smooth muscle actin | Abcam | ab5694 |  |
| Ub(P4D1) | Santa Cruz | SC8017 |  |
| Anti-DYK(M2) agarose bead | SIGMA | A2220 | Used for IP |
| Anti-Myc agarose bead | Thermo Scientific | 20168 | Used for IP |

**Supplemental Table 2. Correlation analyses among *Ttc3* mRNA, *Smurf2* mRNA, and proteins Smurf2, Tgfr1, smad2, and smad3.**

|  | ***Smurf2* mRNA** | **Smurf2 protein** | **Tgfr1** | **Smad2** | **Smad3** |
| --- | --- | --- | --- | --- | --- |
| ***Ttc3* mRNA** | r = -0.351  *P =* 0.182 | r = -0.538  *P =* 0.0314 | r = 0.270  *P =* 0.311 | r = 0.538  *P =* 0.0314 | r = 0.477  *P =* 0.0618 |
| ***Smurf2* mRNA** |  | r = 0.232  *P =* 0.386 | r = 0.155  *P =*0.567 | r = 0.0082  *P =* 0.976 | r = 0.0843  *P =* 0.756 |
| **Smurf2 protein** |  |  | r = -0.561  *P =* 0.0236 | r = -0.806  *P < 0.01* | r = -0.327  *P =* 0.217 |
| **Tgfr1** |  |  |  | r = 0.725  *P <* 0.01 | r = 0.491  *P =* 0.0532 |
| **Smad2** |  |  |  |  | r = 0.603  *P =* 0.0135 |

All the data from both CON and BLEO groups were collected together for correlation analysis. Two numbers in each cell indicate Pearson correlation coefficient (r) and *P* value, respectively. *P* values less than 0.05 are considered as statistically significant. Colors in red and blue show negative and positive correlation, respectively.

**Supplemental Table 3. Correlation analyses among *Ttc3* mRNA and proteins Smurf2, E-cadherin, vimentin, and α-SMA.**

|  | ***Smurf2* mRNA** | **Smurf2 protein** | **E-cadherin** | **Vimentin** | **α-SMA** |
| --- | --- | --- | --- | --- | --- |
| ***Ttc3* mRNA** | r =-0.351  *P =* 0.182 | r = -0.538  *P =* 0.0314 | r = -0.662  *P < 0.01* | r = 0.496  *P =* 0.0507 | r = 0.468  *P =* 0.0677 |
| ***Smurf2* mRNA** |  | r = 0.232  *P =* 0.386 | r = 0.483  *P =* 0.0578 | r = -0.165  *P =* 0.541 | r = -0.105  *P =* 0.699 |
| **Smurf2 protein** |  |  | r = 0.899  *P < 0.01* | r = -0.735  *P < 0.01* | r = -0.885  *P < 0.01* |
| **E-cadherin** |  |  |  | r = -0.782  *P < 0.01* | r = -0.841  *P < 0.01* |
| **Vimentin** |  |  |  |  | r = 0.848  *P < 0.01* |

All the data from both CON and BLEO groups were collected together for correlation analysis. Two numbers in each cell indicate Pearson correlation coefficient (r) and *P* value, respectively. *P* values less than 0.05 are considered as statistically significant. Colors in red and blue show negative and positive correlation, respectively.

**Supplemental References**

1 Kavsak, P. *et al.* Smad7 binds to Smurf2 to form an E3 ubiquitin ligase that targets the TGF beta receptor for degradation. *Mol. Cell* **6**, 1365-1375 (2000).

2 Kim, S. Y. *et al.* Cigarette smoke induces Akt protein degradation by the ubiquitin-proteasome system. *J. Biol. Chem.* **286**, 31932-31943 (2011).

3 Zhao, H. J. *et al.* Bone morphogenetic protein 2 promotes human trophoblast cell invasion by upregulating N-cadherin via non-canonical SMAD2/3 signaling. *Cell Death Dis.* **9**, 174 (2018).

4 Lonn, P. *et al.* Transcriptional induction of salt-inducible kinase 1 by transforming growth factor beta leads to negative regulation of type I receptor signaling in cooperation with the Smurf2 ubiquitin ligase. *J. Biol. Chem.* **287**, 12867-12878 (2012).

5 Uchida, H. *et al.* Histone deacetylase inhibitors stimulate cell migration in human endometrial adenocarcinoma cells through up-regulation of glycodelin. *Endocrinology* **148**, 896-902 (2007).

6 Huh, J. W. *et al.* Bone marrow cells repair cigarette smoke-induced emphysema in rats. *Am. J. Physiol. Lung Cell Mol. Physiol.* **301**, L255-266 (2011).

7 Sato, E., Koyama, S. & Robbins, R. A. Bleomycin stimulates lung fibroblast and epithelial cell lines to release eosinophil chemotactic activity. *Eur. Respir. J.* **16**, 951-958 (2000).
