# Supplementary material for: TTC3 contributes to TGF-β1-induced epithelial−mesenchymal transition and myofibroblast differentiation, potentially through SMURF2 ubiquitylation and degradation
Source: Cell Death Dis. 2019 Jan 29;10(2):92. doi: 10.1038/s41419-019-1308-8 (PMC6351531; doi:10.1038/s41419-019-1308-8)
Supplement: Supplementary file 2 — Supplemental figures [file 41419_2019_1308_MOESM2_ESM.pptx]

## Slide 1
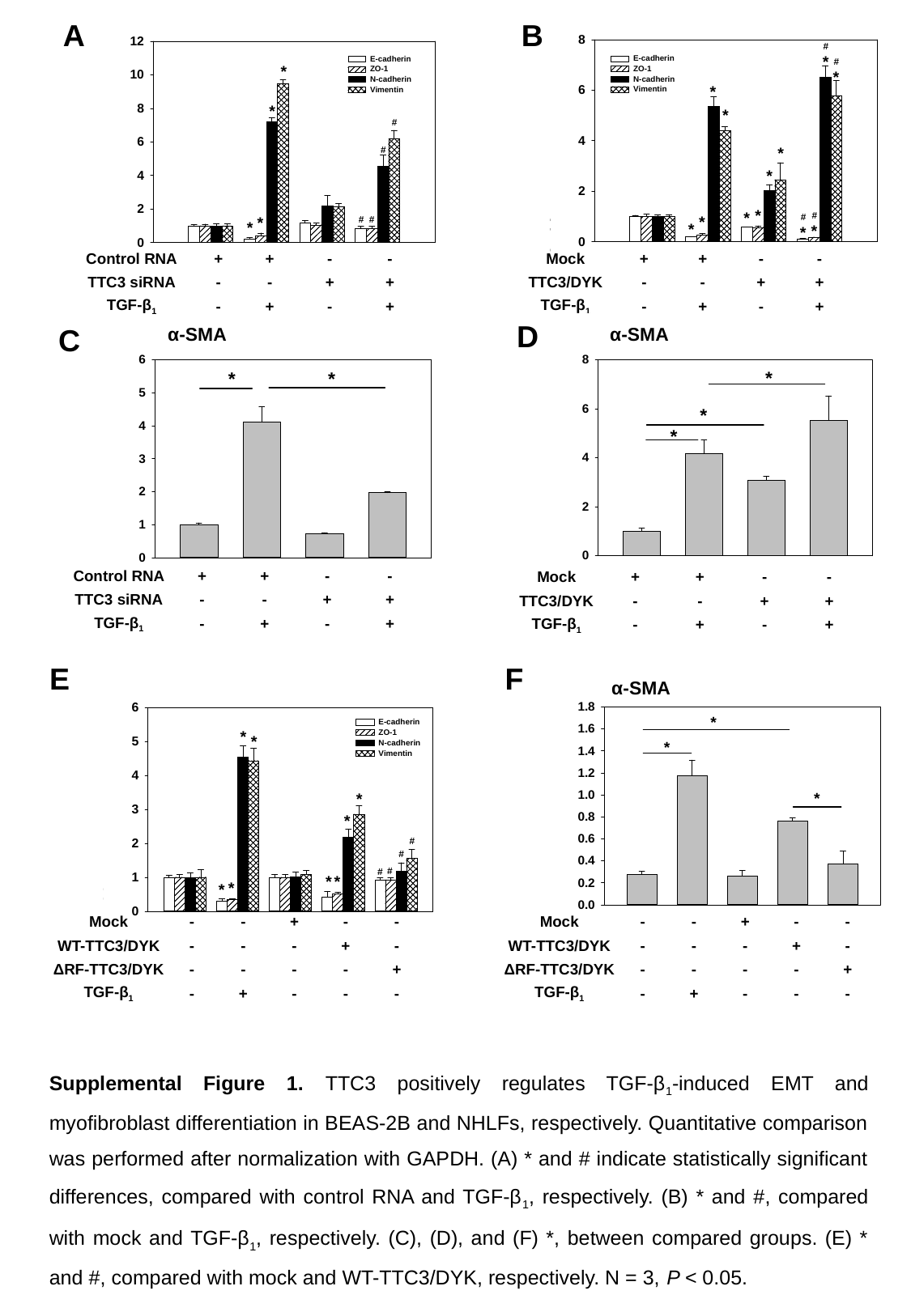

A
B
| Control RNA | + | + | - | - |
| --- | --- | --- | --- | --- |
| TTC3 siRNA | - | - | + | + |
| TGF-β1 | - | + | - | + |
| Mock | + | + | - | - |
| --- | --- | --- | --- | --- |
| TTC3/DYK | - | - | + | + |
| TGF-β1 | - | + | - | + |
D
C
α-SMA
α-SMA
α-SMA
| Control RNA | + | + | - | - |
| --- | --- | --- | --- | --- |
| TTC3 siRNA | - | - | + | + |
| TGF-β1 | - | + | - | + |
| Mock | + | + | - | - |
| --- | --- | --- | --- | --- |
| TTC3/DYK | - | - | + | + |
| TGF-β1 | - | + | - | + |
E
F
α-SMA
| Mock | - | - | + | - | - |
| --- | --- | --- | --- | --- | --- |
| WT-TTC3/DYK | - | - | - | + | - |
| ΔRF-TTC3/DYK | - | - | - | - | + |
| TGF-β1 | - | + | - | - | - |
| Mock | - | - | + | - | - |
| --- | --- | --- | --- | --- | --- |
| WT-TTC3/DYK | - | - | - | + | - |
| ΔRF-TTC3/DYK | - | - | - | - | + |
| TGF-β1 | - | + | - | - | - |
Supplemental Figure 1. TTC3 positively regulates TGF-β1-induced EMT and myofibroblast differentiation in BEAS-2B and NHLFs, respectively. Quantitative comparison was performed after normalization with GAPDH. (A) * and # indicate statistically significant differences, compared with control RNA and TGF-β1, respectively. (B) * and #, compared with mock and TGF-β1, respectively. (C), (D), and (F) *, between compared groups. (E) * and #, compared with mock and WT-TTC3/DYK, respectively. N = 3, P < 0.05.

## Slide 2
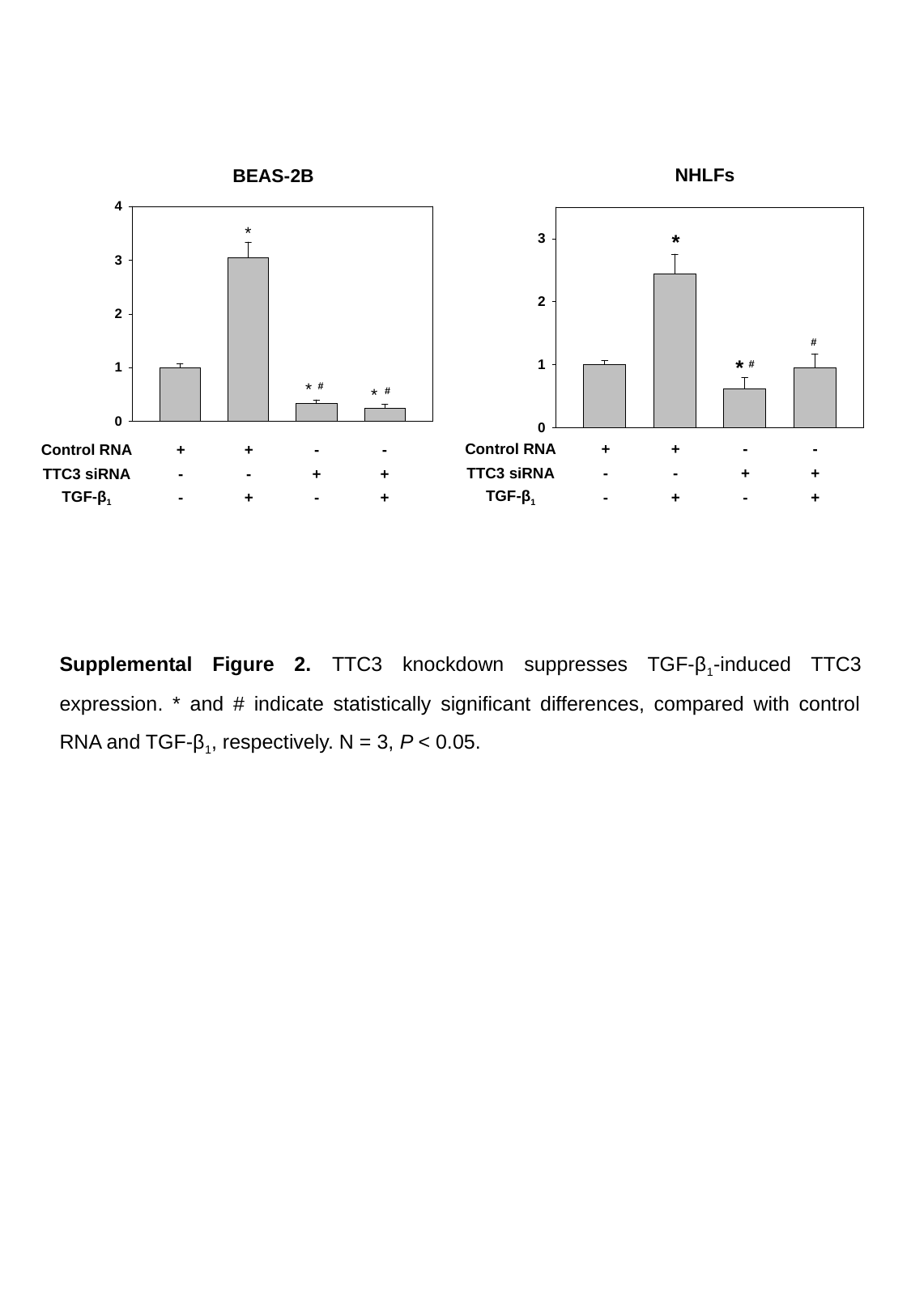

NHLFs
BEAS-2B
| Control RNA | + | + | - | - |
| --- | --- | --- | --- | --- |
| TTC3 siRNA | - | - | + | + |
| TGF-β1 | - | + | - | + |
| Control RNA | + | + | - | - |
| --- | --- | --- | --- | --- |
| TTC3 siRNA | - | - | + | + |
| TGF-β1 | - | + | - | + |
Supplemental Figure 2. TTC3 knockdown suppresses TGF-β1-induced TTC3 expression. * and # indicate statistically significant differences, compared with control RNA and TGF-β1, respectively. N = 3, P < 0.05.

## Slide 3
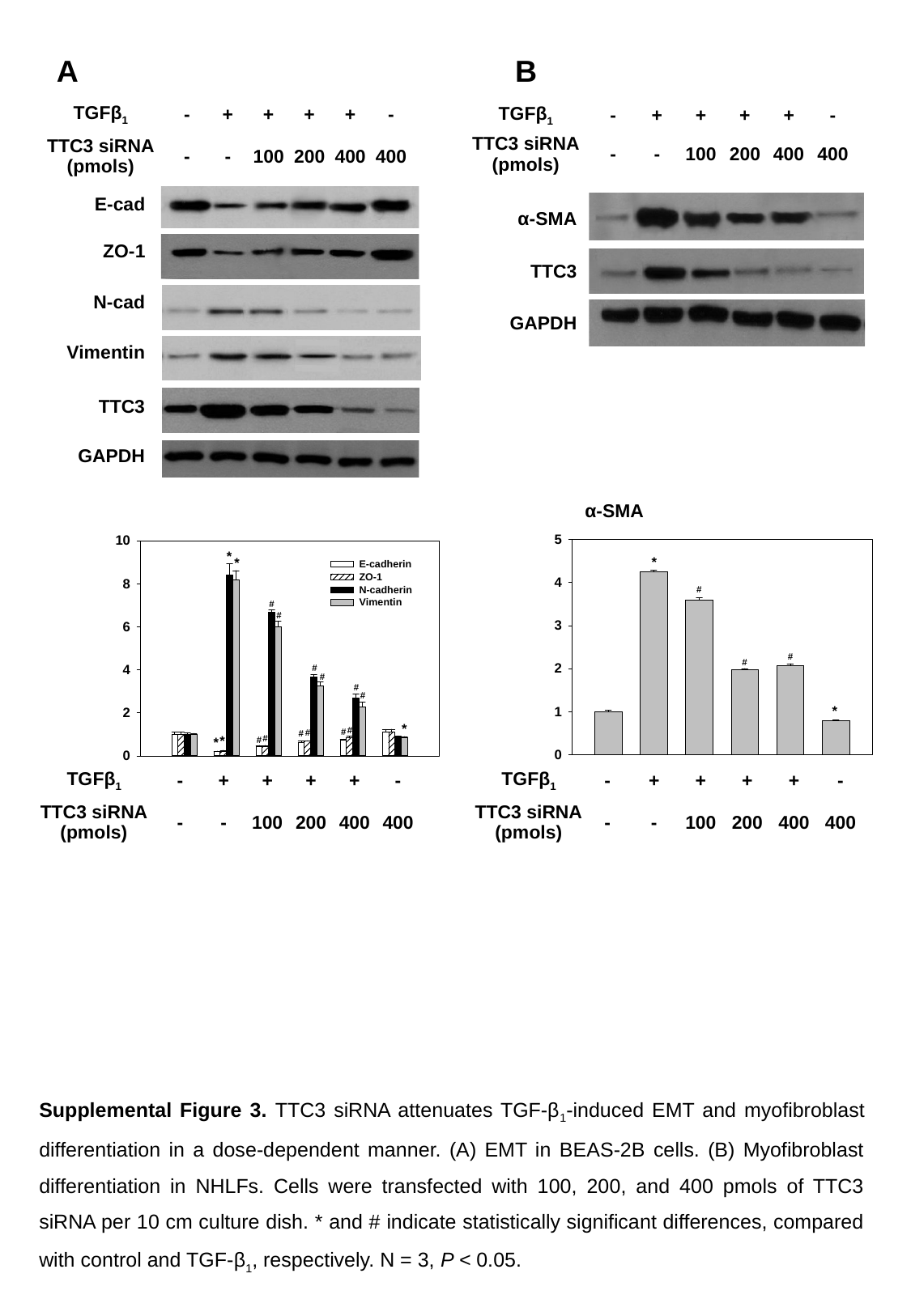

A
B
| TGFβ1 | - | + | + | + | + | - |
| --- | --- | --- | --- | --- | --- | --- |
| TTC3 siRNA (pmols) | - | - | 100 | 200 | 400 | 400 |
| TGFβ1 | - | + | + | + | + | - |
| --- | --- | --- | --- | --- | --- | --- |
| TTC3 siRNA (pmols) | - | - | 100 | 200 | 400 | 400 |
E-cad
α-SMA
ZO-1
TTC3
N-cad
GAPDH
Vimentin
TTC3
GAPDH
α-SMA
| TGFβ1 | - | + | + | + | + | - |
| --- | --- | --- | --- | --- | --- | --- |
| TTC3 siRNA (pmols) | - | - | 100 | 200 | 400 | 400 |
| TGFβ1 | - | + | + | + | + | - |
| --- | --- | --- | --- | --- | --- | --- |
| TTC3 siRNA (pmols) | - | - | 100 | 200 | 400 | 400 |
Supplemental Figure 3. TTC3 siRNA attenuates TGF-β1-induced EMT and myofibroblast differentiation in a dose-dependent manner. (A) EMT in BEAS-2B cells. (B) Myofibroblast differentiation in NHLFs. Cells were transfected with 100, 200, and 400 pmols of TTC3 siRNA per 10 cm culture dish. * and # indicate statistically significant differences, compared with control and TGF-β1, respectively. N = 3, P < 0.05.

## Slide 4
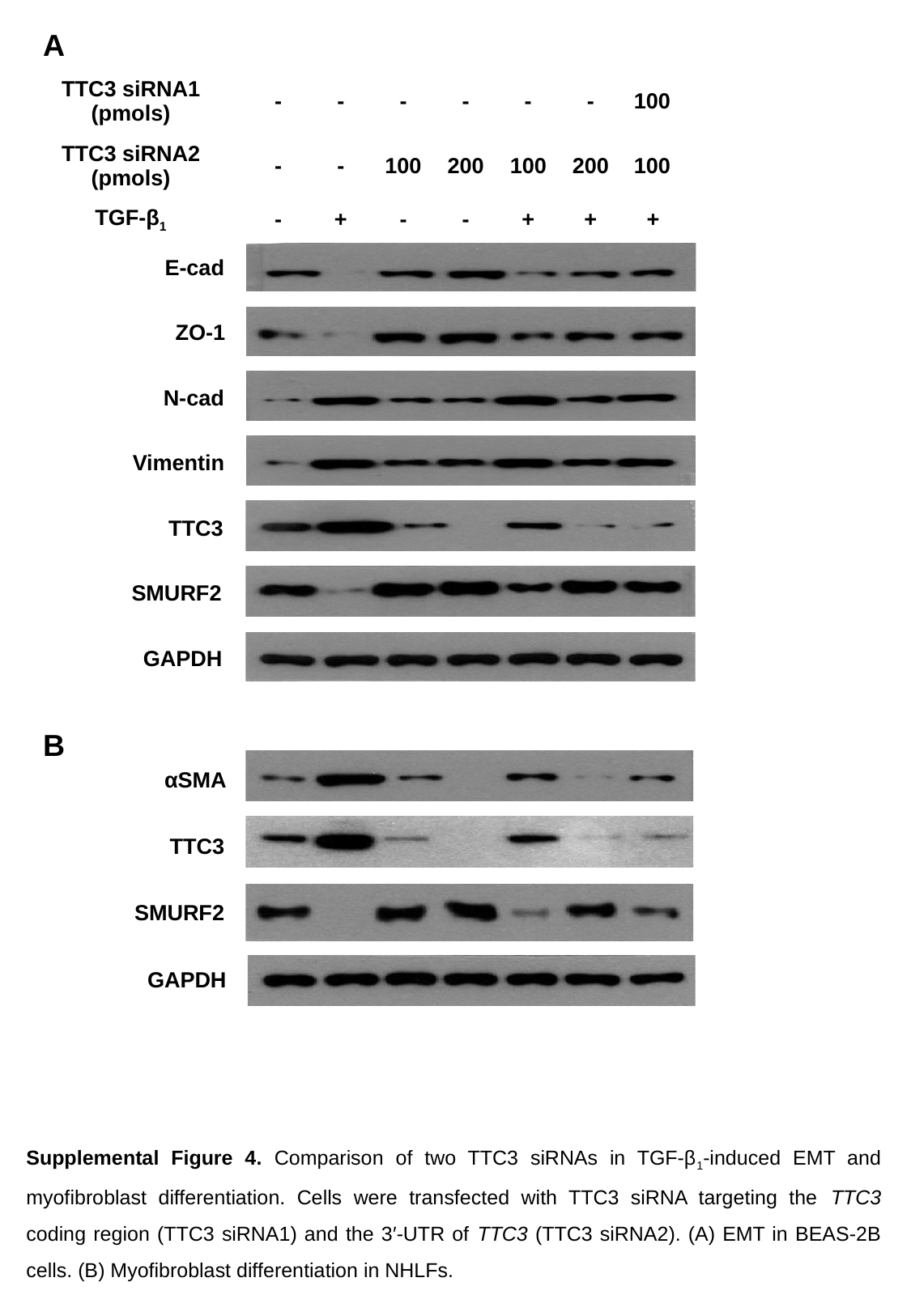

A
| TTC3 siRNA1 (pmols) | - | - | - | - | - | - | 100 |
| --- | --- | --- | --- | --- | --- | --- | --- |
| TTC3 siRNA2 (pmols) | - | - | 100 | 200 | 100 | 200 | 100 |
| TGF-β1 | - | + | - | - | + | + | + |
E-cad
ZO-1
N-cad
Vimentin
TTC3
SMURF2
GAPDH
B
αSMA
TTC3
SMURF2
GAPDH
Supplemental Figure 4. Comparison of two TTC3 siRNAs in TGF-β1-induced EMT and myofibroblast differentiation. Cells were transfected with TTC3 siRNA targeting the TTC3 coding region (TTC3 siRNA1) and the 3′-UTR of TTC3 (TTC3 siRNA2). (A) EMT in BEAS-2B cells. (B) Myofibroblast differentiation in NHLFs.

## Slide 5
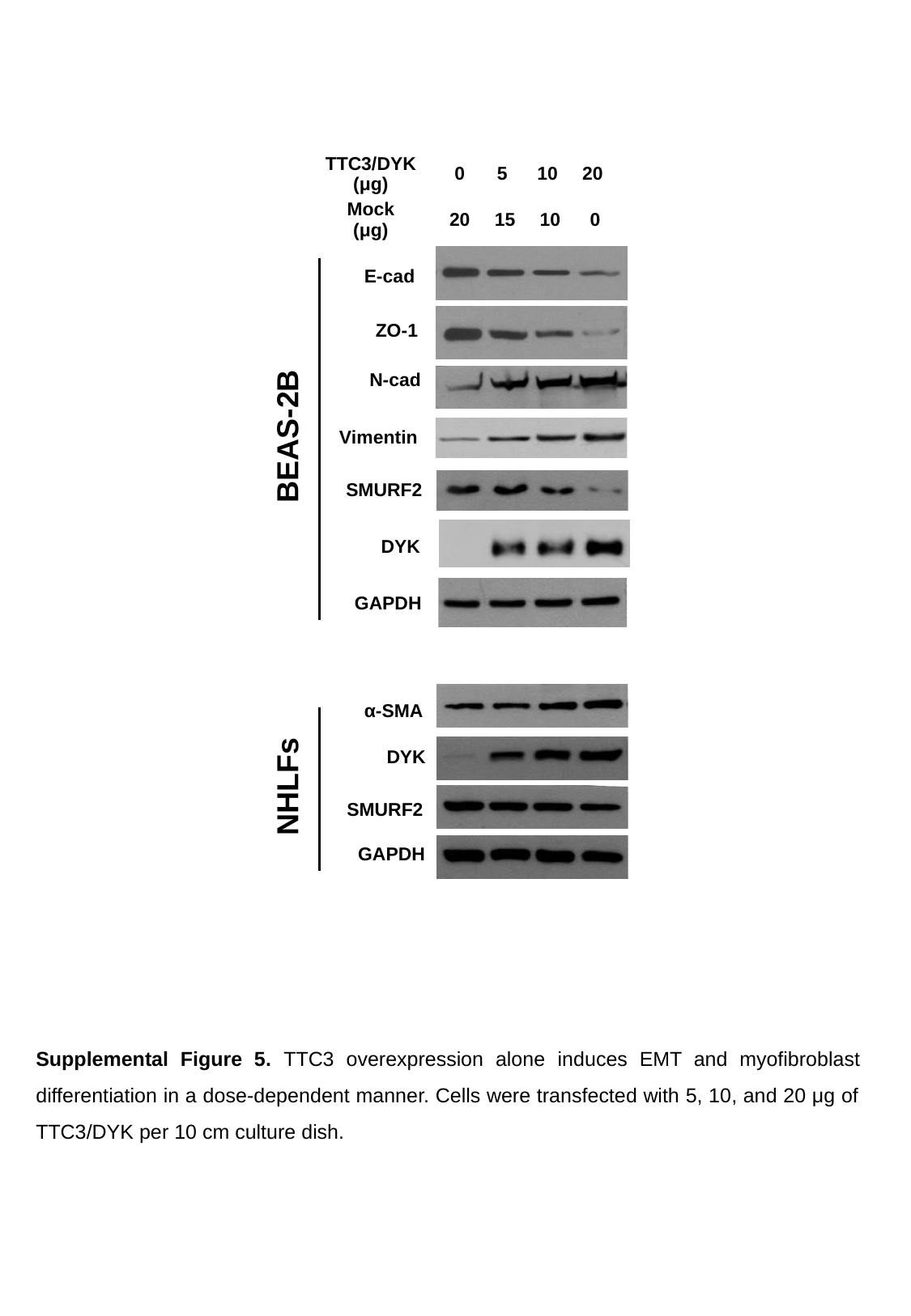

| TTC3/DYK (μg) | 0 | 5 | 10 | 20 |
| --- | --- | --- | --- | --- |
| Mock (μg) | 20 | 15 | 10 | 0 |
E-cad
ZO-1
N-cad
Vimentin
SMURF2
DYK
GAPDH
BEAS-2B
α-SMA
DYK
NHLFs
SMURF2
GAPDH
Supplemental Figure 5. TTC3 overexpression alone induces EMT and myofibroblast differentiation in a dose-dependent manner. Cells were transfected with 5, 10, and 20 μg of TTC3/DYK per 10 cm culture dish.

## Slide 6
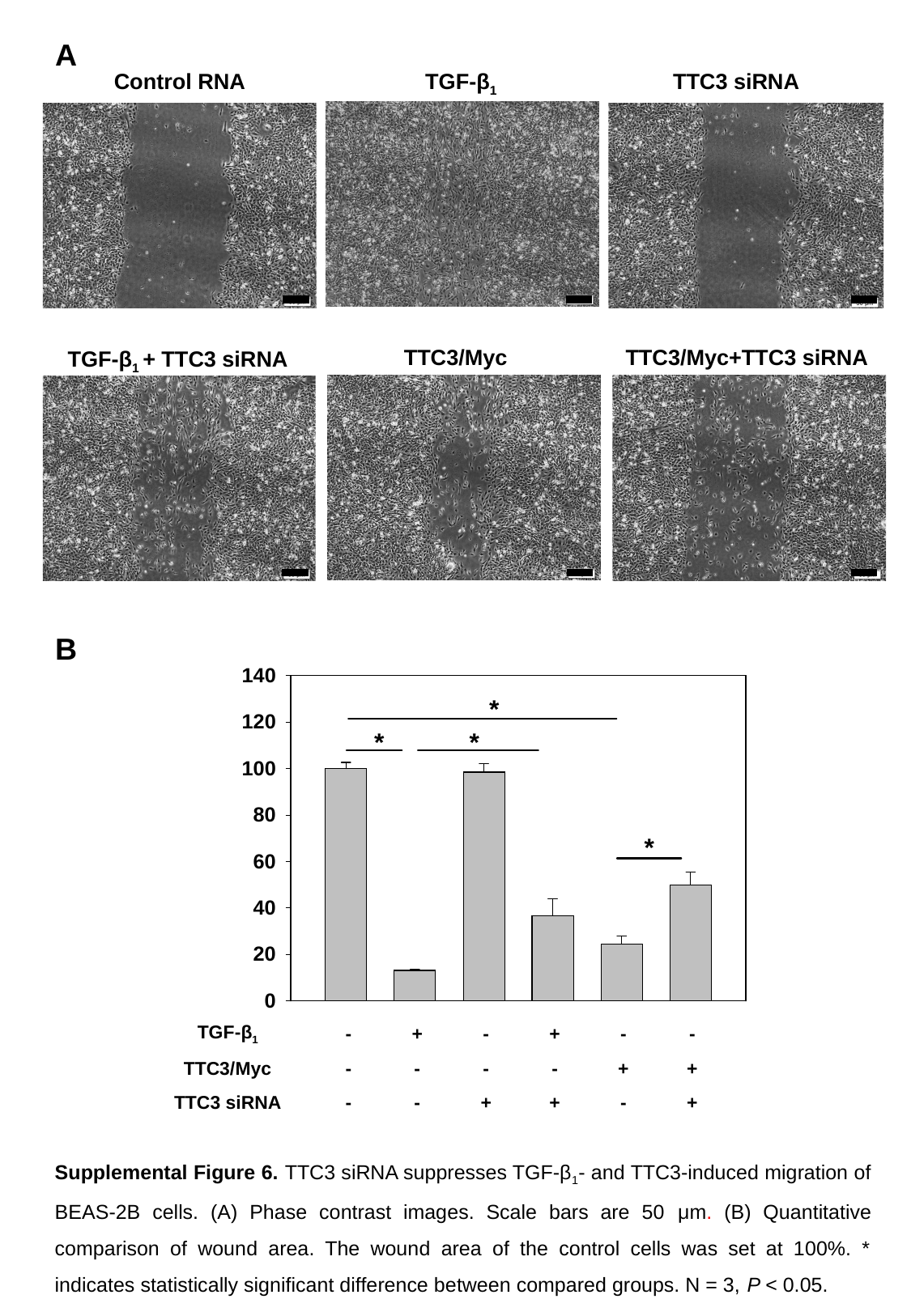

A
Control RNA
TGF-β1
TTC3 siRNA
TTC3/Myc
TTC3/Myc+TTC3 siRNA
TGF-β1 + TTC3 siRNA
B
| TGF-β1 | - | + | - | + | - | - |
| --- | --- | --- | --- | --- | --- | --- |
| TTC3/Myc | - | - | - | - | + | + |
| TTC3 siRNA | - | - | + | + | - | + |
Supplemental Figure 6. TTC3 siRNA suppresses TGF-β1- and TTC3-induced migration of BEAS-2B cells. (A) Phase contrast images. Scale bars are 50 μm. (B) Quantitative comparison of wound area. The wound area of the control cells was set at 100%. * indicates statistically significant difference between compared groups. N = 3, P < 0.05.

## Slide 7
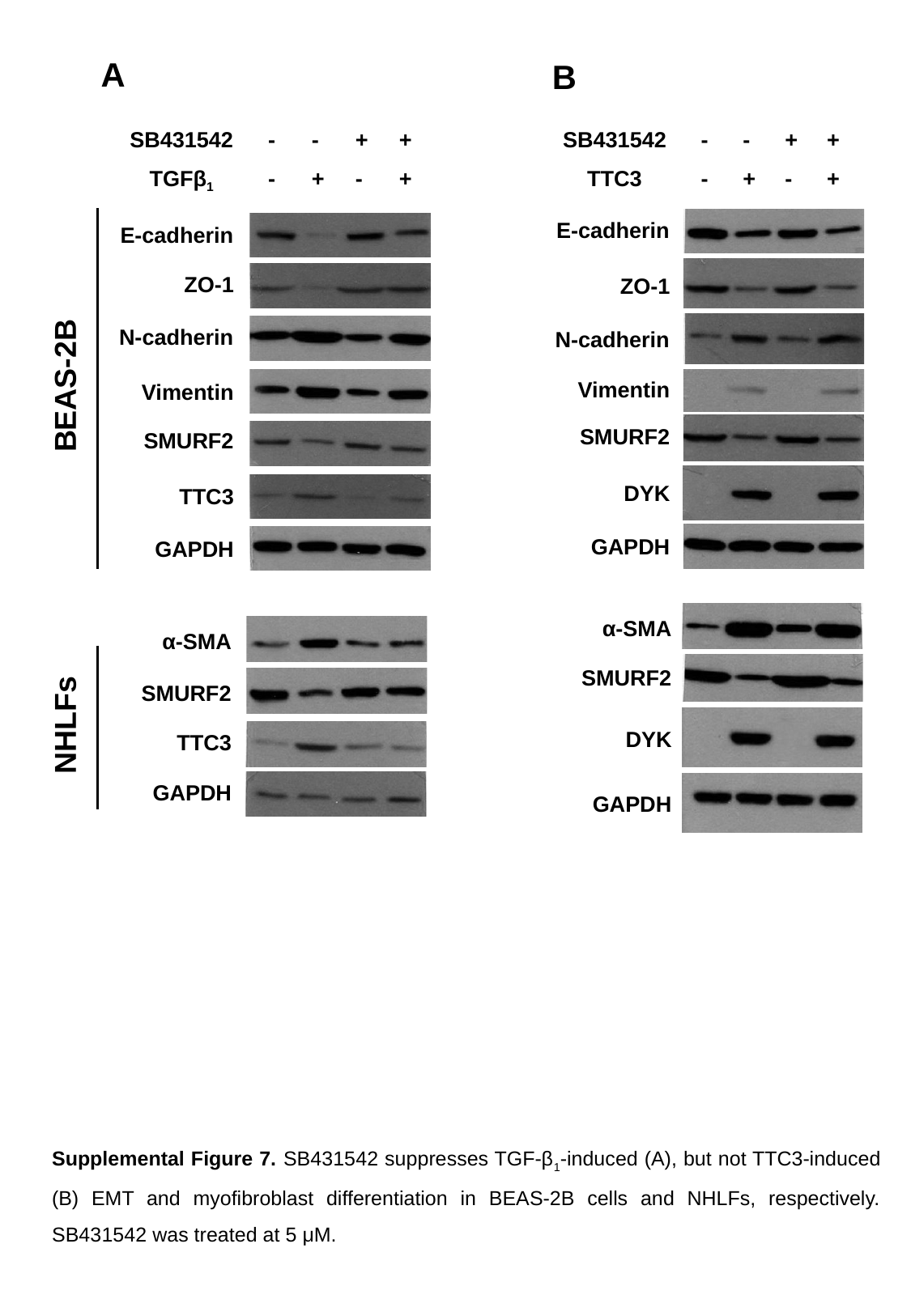

A
B
| SB431542 | - | - | + | + |
| --- | --- | --- | --- | --- |
| TGFβ1 | - | + | - | + |
| SB431542 | - | - | + | + |
| --- | --- | --- | --- | --- |
| TTC3 | - | + | - | + |
E-cadherin
E-cadherin
ZO-1
ZO-1
N-cadherin
N-cadherin
BEAS-2B
Vimentin
Vimentin
SMURF2
SMURF2
DYK
TTC3
GAPDH
GAPDH
α-SMA
α-SMA
SMURF2
SMURF2
NHLFs
DYK
TTC3
GAPDH
GAPDH
Supplemental Figure 7. SB431542 suppresses TGF-β1-induced (A), but not TTC3-induced (B) EMT and myofibroblast differentiation in BEAS-2B cells and NHLFs, respectively. SB431542 was treated at 5 μM.

## Slide 8
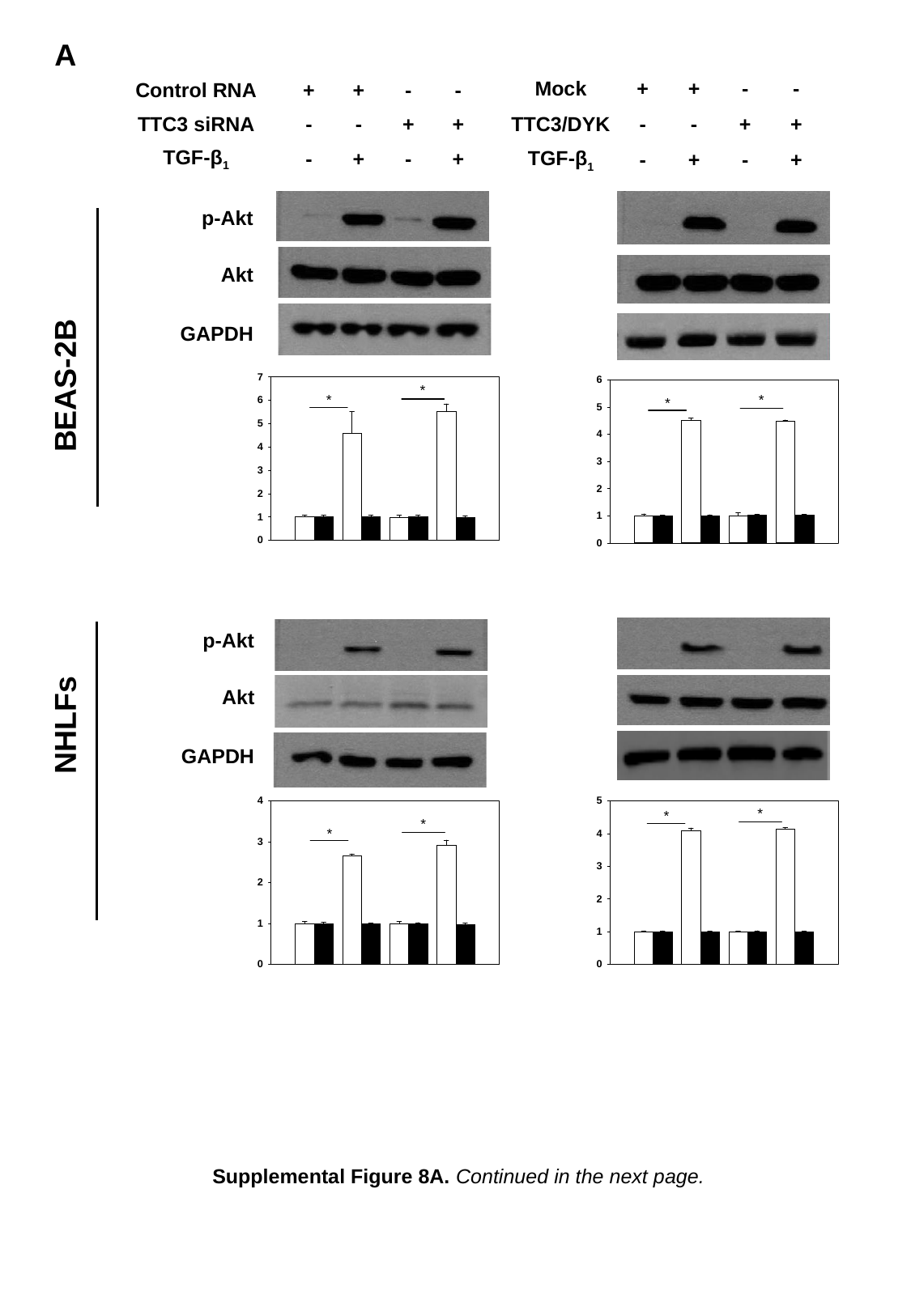

A
| Mock | + | + | - | - |
| --- | --- | --- | --- | --- |
| TTC3/DYK | - | - | + | + |
| TGF-β1 | - | + | - | + |
| Control RNA | + | + | - | - |
| --- | --- | --- | --- | --- |
| TTC3 siRNA | - | - | + | + |
| TGF-β1 | - | + | - | + |
p-Akt
Akt
GAPDH
BEAS-2B
p-Akt
Akt
NHLFs
GAPDH
Supplemental Figure 8A. Continued in the next page.

## Slide 9
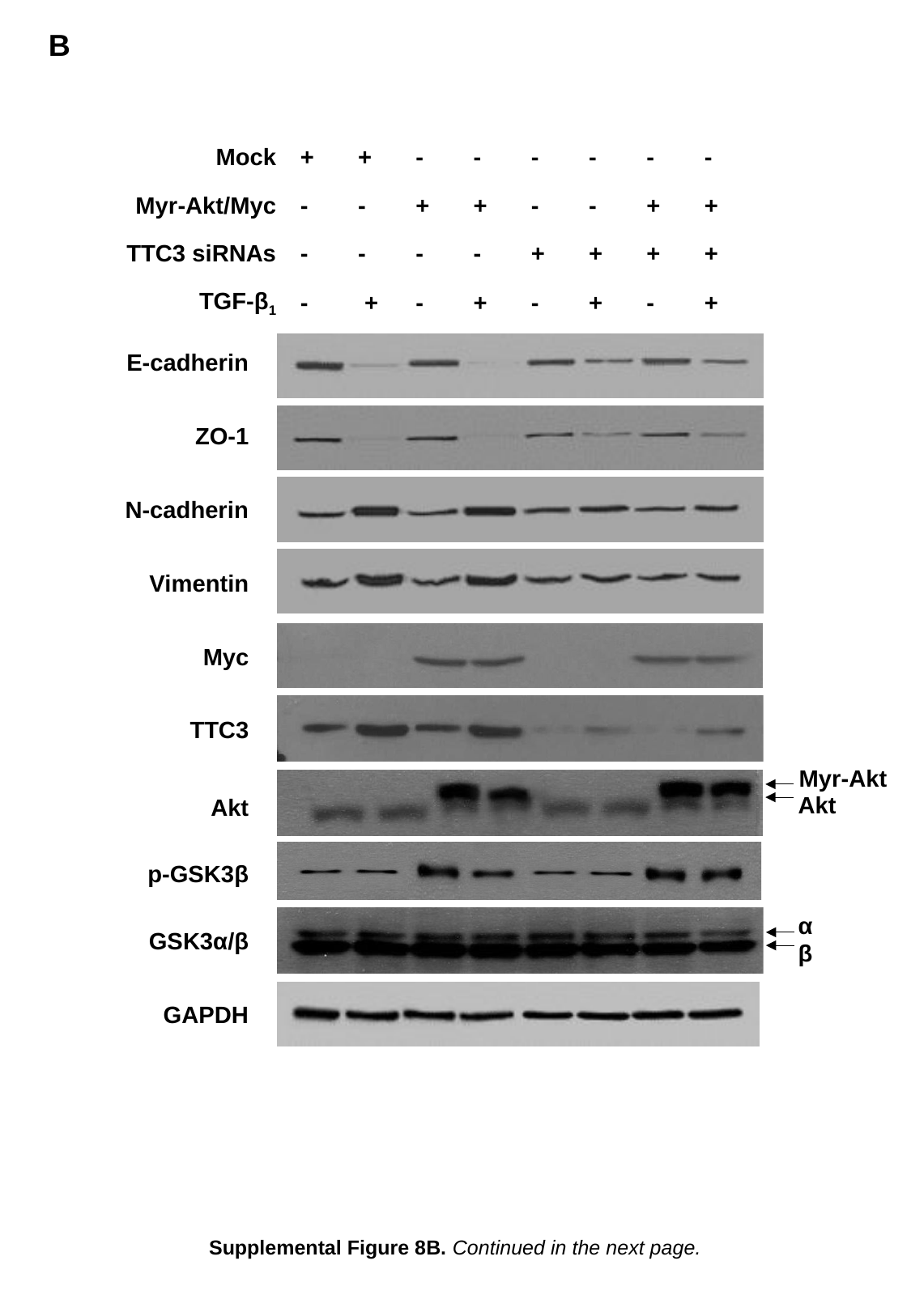

B
| |
| --- |
| |
| |
| |
| Mock | + | + | - | - | - | - | - | - |
| --- | --- | --- | --- | --- | --- | --- | --- | --- |
| Myr-Akt/Myc | - | - | + | + | - | - | + | + |
| TTC3 siRNAs | - | - | - | - | + | + | + | + |
| TGF-β1 | - | + | - | + | - | + | - | + |
E-cadherin
ZO-1
N-cadherin
Vimentin
Myc
TTC3
Myr-Akt
Akt
Akt
p-GSK3β
α
GSK3α/β
β
GAPDH
Supplemental Figure 8B. Continued in the next page.

## Slide 10
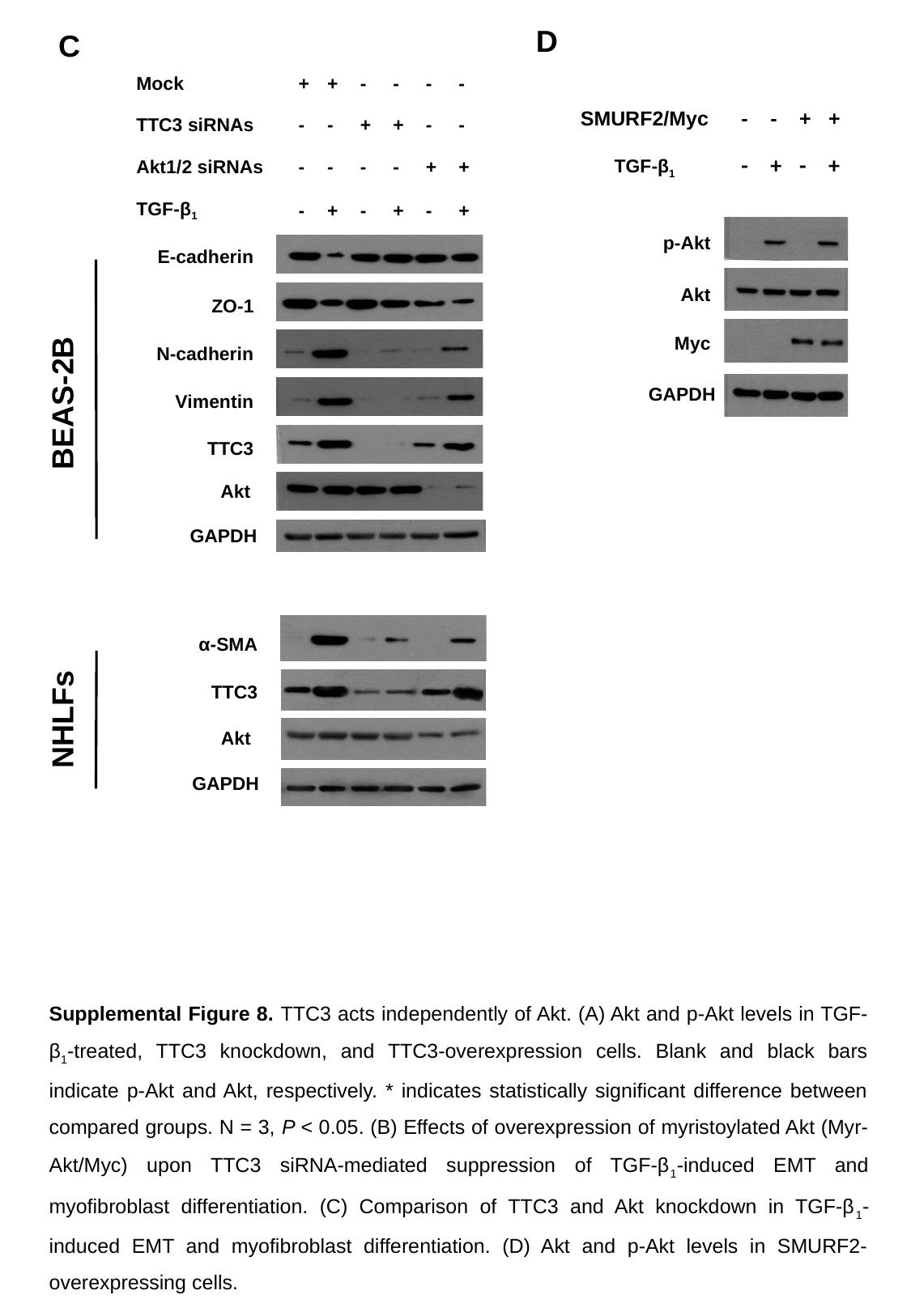

D
C
| Mock | + | + | - | - | - | - |
| --- | --- | --- | --- | --- | --- | --- |
| TTC3 siRNAs | - | - | + | + | - | - |
| Akt1/2 siRNAs | - | - | - | - | + | + |
| TGF-β1 | - | + | - | + | - | + |
| SMURF2/Myc | - | - | + | + |
| --- | --- | --- | --- | --- |
| TGF-β1 | - | + | - | + |
p-Akt
Akt
Myc
GAPDH
E-cadherin
ZO-1
N-cadherin
BEAS-2B
Vimentin
TTC3
Akt
GAPDH
α-SMA
TTC3
NHLFs
Akt
GAPDH
Supplemental Figure 8. TTC3 acts independently of Akt. (A) Akt and p-Akt levels in TGF-β1-treated, TTC3 knockdown, and TTC3-overexpression cells. Blank and black bars indicate p-Akt and Akt, respectively. * indicates statistically significant difference between compared groups. N = 3, P < 0.05. (B) Effects of overexpression of myristoylated Akt (Myr-Akt/Myc) upon TTC3 siRNA-mediated suppression of TGF-β1-induced EMT and myofibroblast differentiation. (C) Comparison of TTC3 and Akt knockdown in TGF-β1-induced EMT and myofibroblast differentiation. (D) Akt and p-Akt levels in SMURF2-overexpressing cells.

## Slide 11
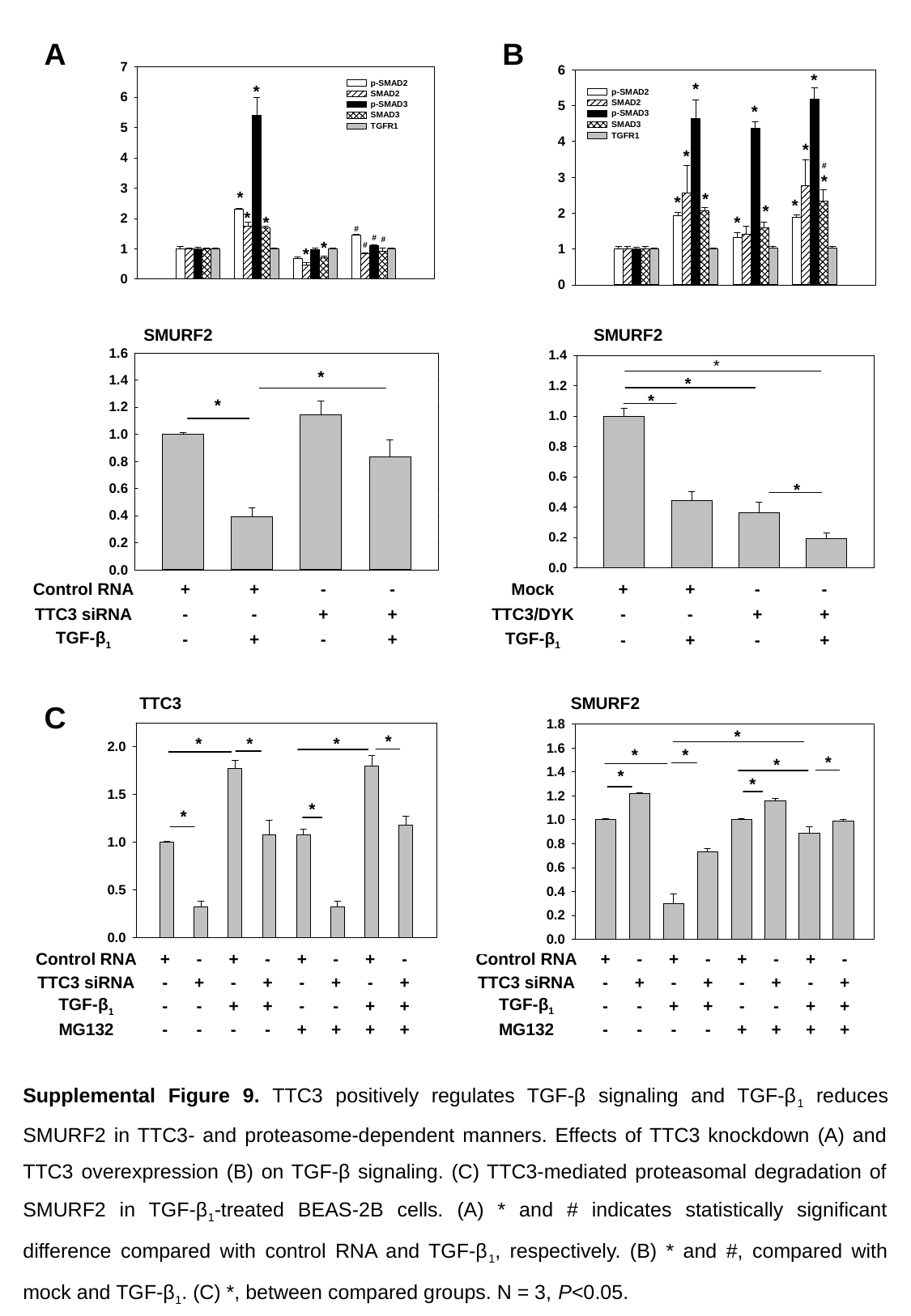

A
B
SMURF2
SMURF2
SMURF2
| Control RNA | + | + | - | - |
| --- | --- | --- | --- | --- |
| TTC3 siRNA | - | - | + | + |
| TGF-β1 | - | + | - | + |
| Mock | + | + | - | - |
| --- | --- | --- | --- | --- |
| TTC3/DYK | - | - | + | + |
| TGF-β1 | - | + | - | + |
TTC3
SMURF2
C
| Control RNA | + | - | + | - | + | - | + | - |
| --- | --- | --- | --- | --- | --- | --- | --- | --- |
| TTC3 siRNA | - | + | - | + | - | + | - | + |
| TGF-β1 | - | - | + | + | - | - | + | + |
| MG132 | - | - | - | - | + | + | + | + |
| Control RNA | + | - | + | - | + | - | + | - |
| --- | --- | --- | --- | --- | --- | --- | --- | --- |
| TTC3 siRNA | - | + | - | + | - | + | - | + |
| TGF-β1 | - | - | + | + | - | - | + | + |
| MG132 | - | - | - | - | + | + | + | + |
Supplemental Figure 9. TTC3 positively regulates TGF-β signaling and TGF-β1 reduces SMURF2 in TTC3- and proteasome-dependent manners. Effects of TTC3 knockdown (A) and TTC3 overexpression (B) on TGF-β signaling. (C) TTC3-mediated proteasomal degradation of SMURF2 in TGF-β1-treated BEAS-2B cells. (A) * and # indicates statistically significant difference compared with control RNA and TGF-β1, respectively. (B) * and #, compared with mock and TGF-β1. (C) *, between compared groups. N = 3, P<0.05.

## Slide 12
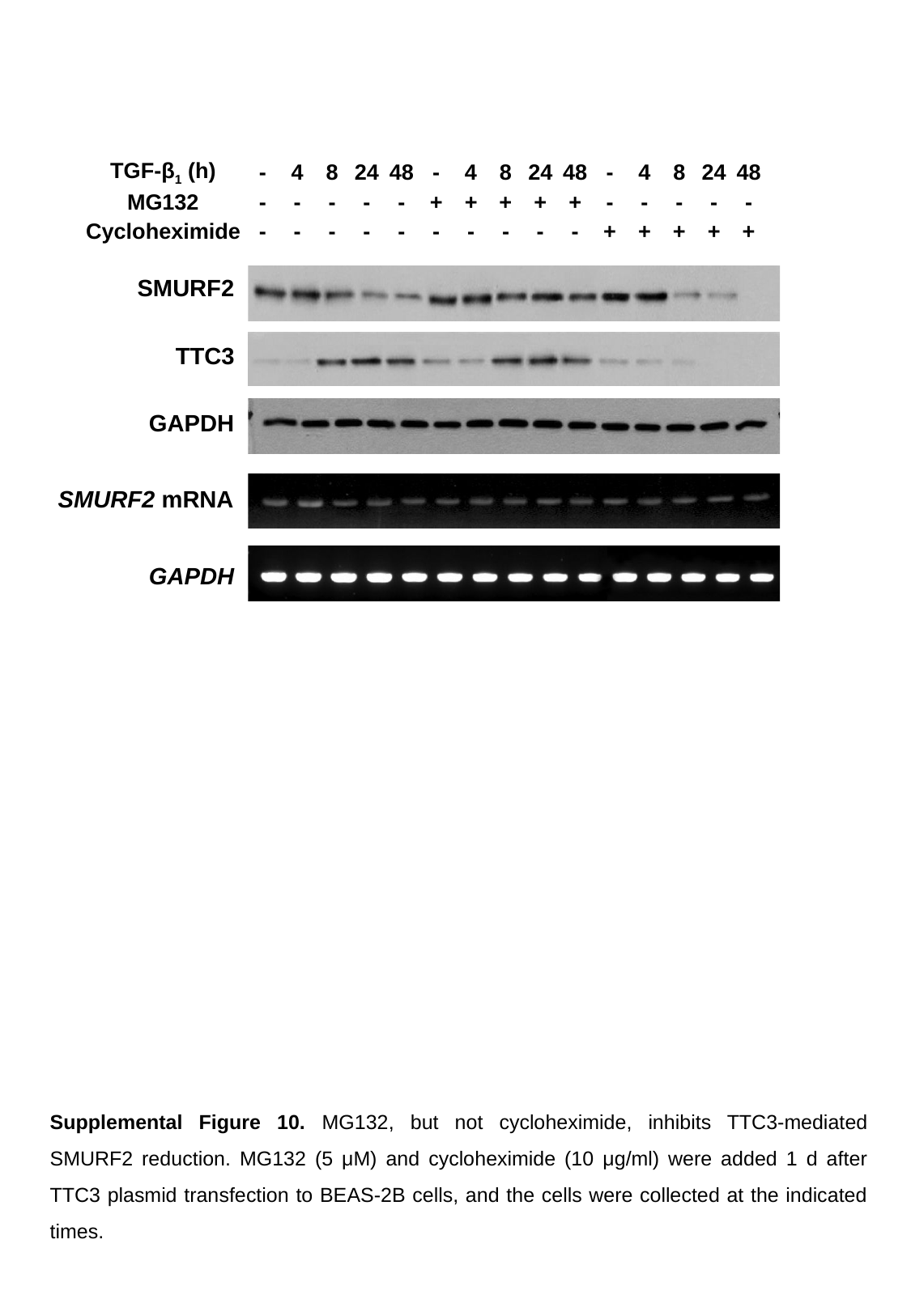

| TGF-β1 (h) | - | 4 | 8 | 24 | 48 | - | 4 | 8 | 24 | 48 | - | 4 | 8 | 24 | 48 |
| --- | --- | --- | --- | --- | --- | --- | --- | --- | --- | --- | --- | --- | --- | --- | --- |
| MG132 | - | - | - | - | - | + | + | + | + | + | - | - | - | - | - |
| Cycloheximide | - | - | - | - | - | - | - | - | - | - | + | + | + | + | + |
SMURF2
TTC3
GAPDH
SMURF2 mRNA
GAPDH
Supplemental Figure 10. MG132, but not cycloheximide, inhibits TTC3-mediated SMURF2 reduction. MG132 (5 μM) and cycloheximide (10 μg/ml) were added 1 d after TTC3 plasmid transfection to BEAS-2B cells, and the cells were collected at the indicated times.

## Slide 13
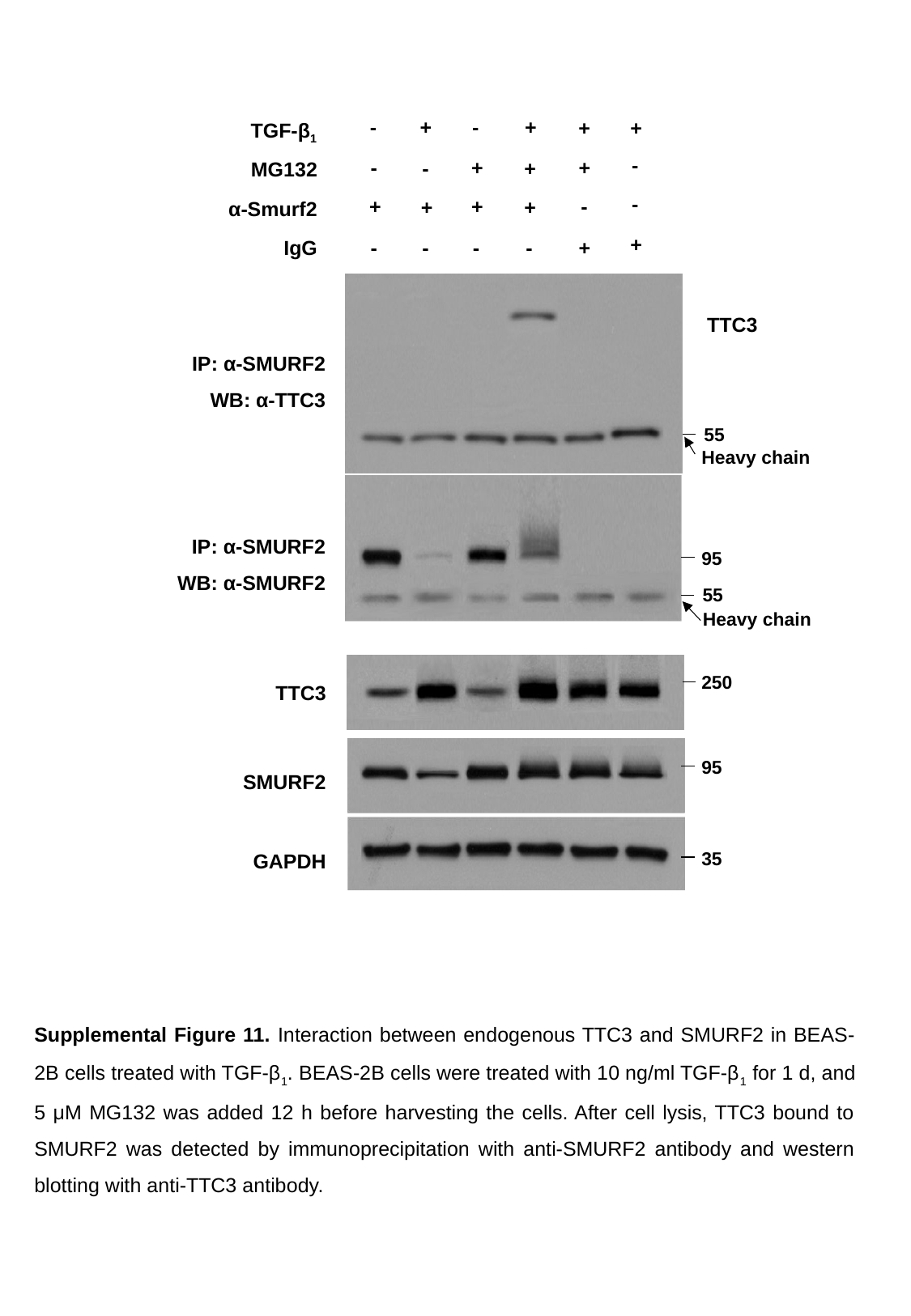

+
+
-
-
+
+
TGF-β1
-
-
+
+
-
+
-
+
+
-
+
+
+
-
+
-
-
-
MG132
α-Smurf2
IgG
TTC3
IP: α-SMURF2
WB: α-TTC3
55
Heavy chain
IP: α-SMURF2
WB: α-SMURF2
95
55
Heavy chain
250
TTC3
95
SMURF2
35
GAPDH
Supplemental Figure 11. Interaction between endogenous TTC3 and SMURF2 in BEAS-2B cells treated with TGF-β1. BEAS-2B cells were treated with 10 ng/ml TGF-β1 for 1 d, and 5 μM MG132 was added 12 h before harvesting the cells. After cell lysis, TTC3 bound to SMURF2 was detected by immunoprecipitation with anti-SMURF2 antibody and western blotting with anti-TTC3 antibody.

## Slide 14
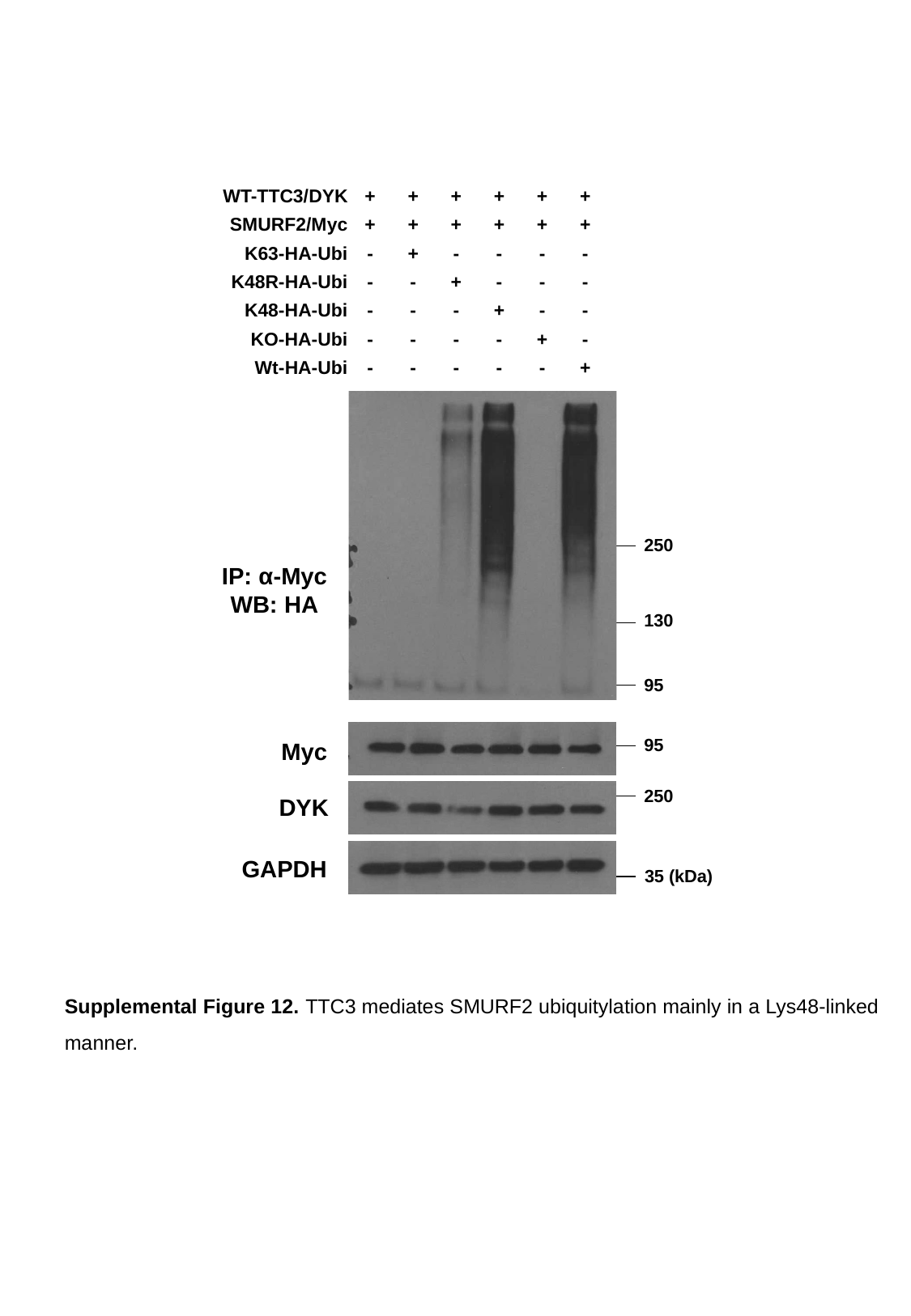

| WT-TTC3/DYK | + | + | + | + | + | + |
| --- | --- | --- | --- | --- | --- | --- |
| SMURF2/Myc | + | + | + | + | + | + |
| K63-HA-Ubi | - | + | - | - | - | - |
| K48R-HA-Ubi | - | - | + | - | - | - |
| K48-HA-Ubi | - | - | - | + | - | - |
| KO-HA-Ubi | - | - | - | - | + | - |
| Wt-HA-Ubi | - | - | - | - | - | + |
250
IP: α-Myc
WB: HA
130
95
95
Myc
250
DYK
GAPDH
35 (kDa)
Supplemental Figure 12. TTC3 mediates SMURF2 ubiquitylation mainly in a Lys48-linked manner.

## Slide 15
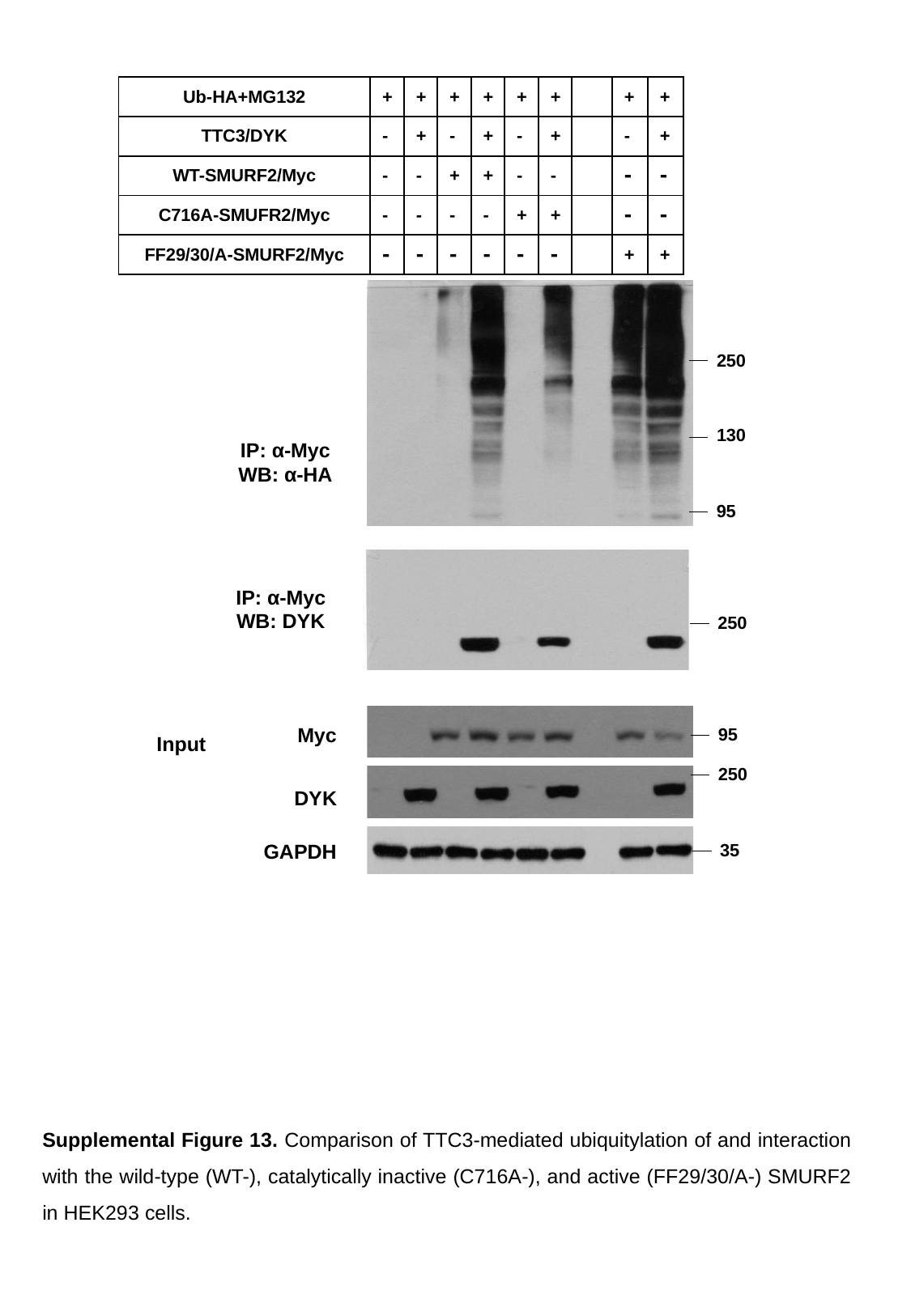

| Ub-HA+MG132 | + | + | + | + | + | + | | + | + |
| --- | --- | --- | --- | --- | --- | --- | --- | --- | --- |
| TTC3/DYK | - | + | - | + | - | + | | - | + |
| WT-SMURF2/Myc | - | - | + | + | - | - | | - | - |
| C716A-SMUFR2/Myc | - | - | - | - | + | + | | - | - |
| FF29/30/A-SMURF2/Myc | - | - | - | - | - | - | | + | + |
250
130
95
IP: α-Myc
WB: α-HA
IP: α-Myc
WB: DYK
250
Myc
95
Input
250
DYK
GAPDH
35
Supplemental Figure 13. Comparison of TTC3-mediated ubiquitylation of and interaction with the wild-type (WT-), catalytically inactive (C716A-), and active (FF29/30/A-) SMURF2 in HEK293 cells.

## Slide 16
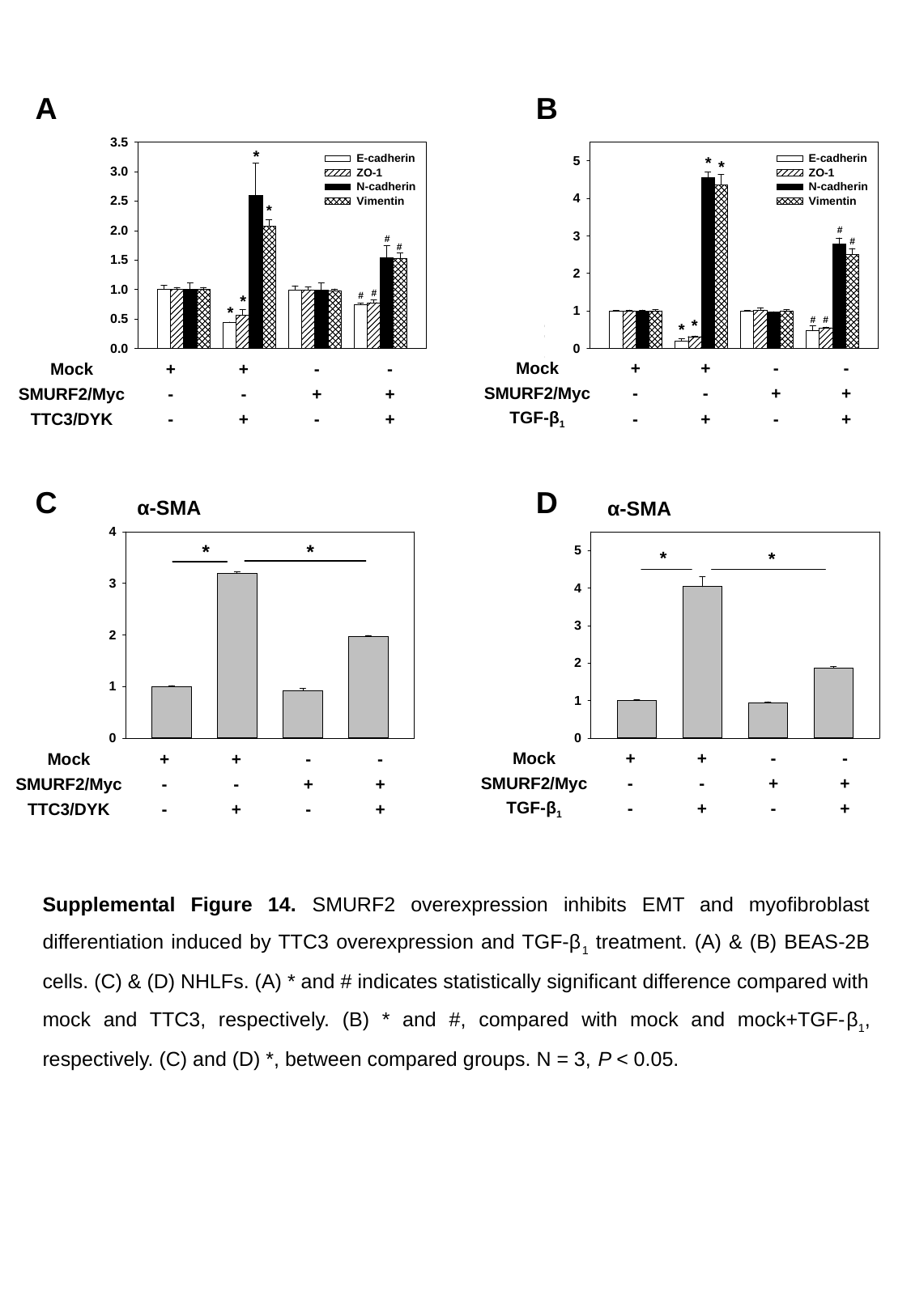

A
B
| Mock | + | + | - | - |
| --- | --- | --- | --- | --- |
| SMURF2/Myc | - | - | + | + |
| TGF-β1 | - | + | - | + |
| Mock | + | + | - | - |
| --- | --- | --- | --- | --- |
| SMURF2/Myc | - | - | + | + |
| TTC3/DYK | - | + | - | + |
C
D
α-SMA
α-SMA
| Mock | + | + | - | - |
| --- | --- | --- | --- | --- |
| SMURF2/Myc | - | - | + | + |
| TGF-β1 | - | + | - | + |
| Mock | + | + | - | - |
| --- | --- | --- | --- | --- |
| SMURF2/Myc | - | - | + | + |
| TTC3/DYK | - | + | - | + |
Supplemental Figure 14. SMURF2 overexpression inhibits EMT and myofibroblast differentiation induced by TTC3 overexpression and TGF-β1 treatment. (A) & (B) BEAS-2B cells. (C) & (D) NHLFs. (A) * and # indicates statistically significant difference compared with mock and TTC3, respectively. (B) * and #, compared with mock and mock+TGF-β1, respectively. (C) and (D) *, between compared groups. N = 3, P < 0.05.

## Slide 17
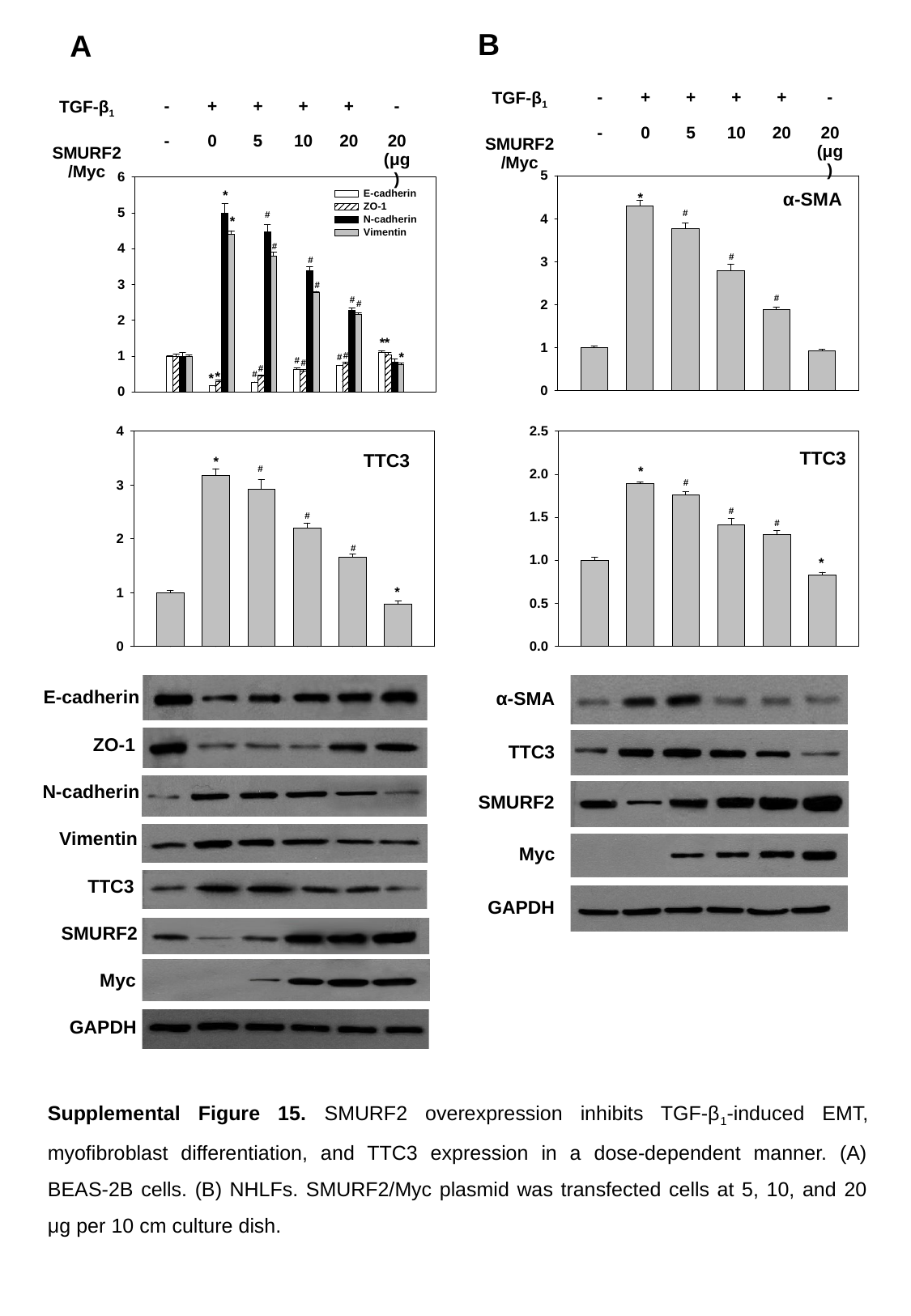

B
A
| TGF-β1 | - | + | + | + | + | - |
| --- | --- | --- | --- | --- | --- | --- |
| SMURF2 /Myc | - | 0 | 5 | 10 | 20 | 20 (μg) |
| TGF-β1 | - | + | + | + | + | - |
| --- | --- | --- | --- | --- | --- | --- |
| SMURF2 /Myc | - | 0 | 5 | 10 | 20 | 20 (μg) |
α-SMA
TTC3
TTC3
E-cadherin
α-SMA
ZO-1
TTC3
N-cadherin
SMURF2
Vimentin
Myc
TTC3
GAPDH
SMURF2
Myc
GAPDH
Supplemental Figure 15. SMURF2 overexpression inhibits TGF-β1-induced EMT, myofibroblast differentiation, and TTC3 expression in a dose-dependent manner. (A) BEAS-2B cells. (B) NHLFs. SMURF2/Myc plasmid was transfected cells at 5, 10, and 20 μg per 10 cm culture dish.

## Slide 18
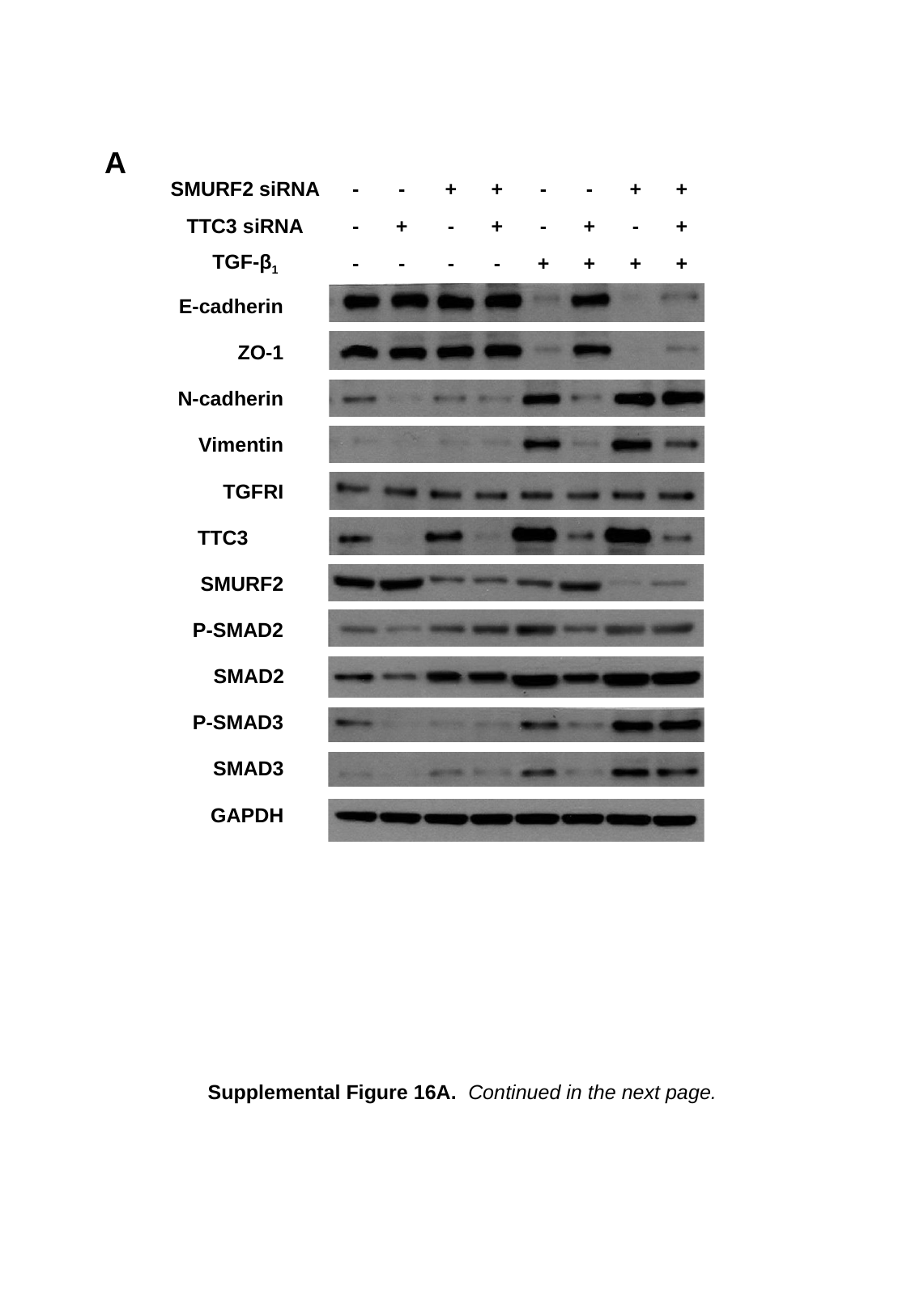

A
| SMURF2 siRNA | - | - | + | + | - | - | + | + |
| --- | --- | --- | --- | --- | --- | --- | --- | --- |
| TTC3 siRNA | - | + | - | + | - | + | - | + |
| TGF-β1 | - | - | - | - | + | + | + | + |
E-cadherin
ZO-1
N-cadherin
Vimentin
TGFRI
TTC3
SMURF2
P-SMAD2
SMAD2
P-SMAD3
SMAD3
GAPDH
Supplemental Figure 16A. Continued in the next page.

## Slide 19
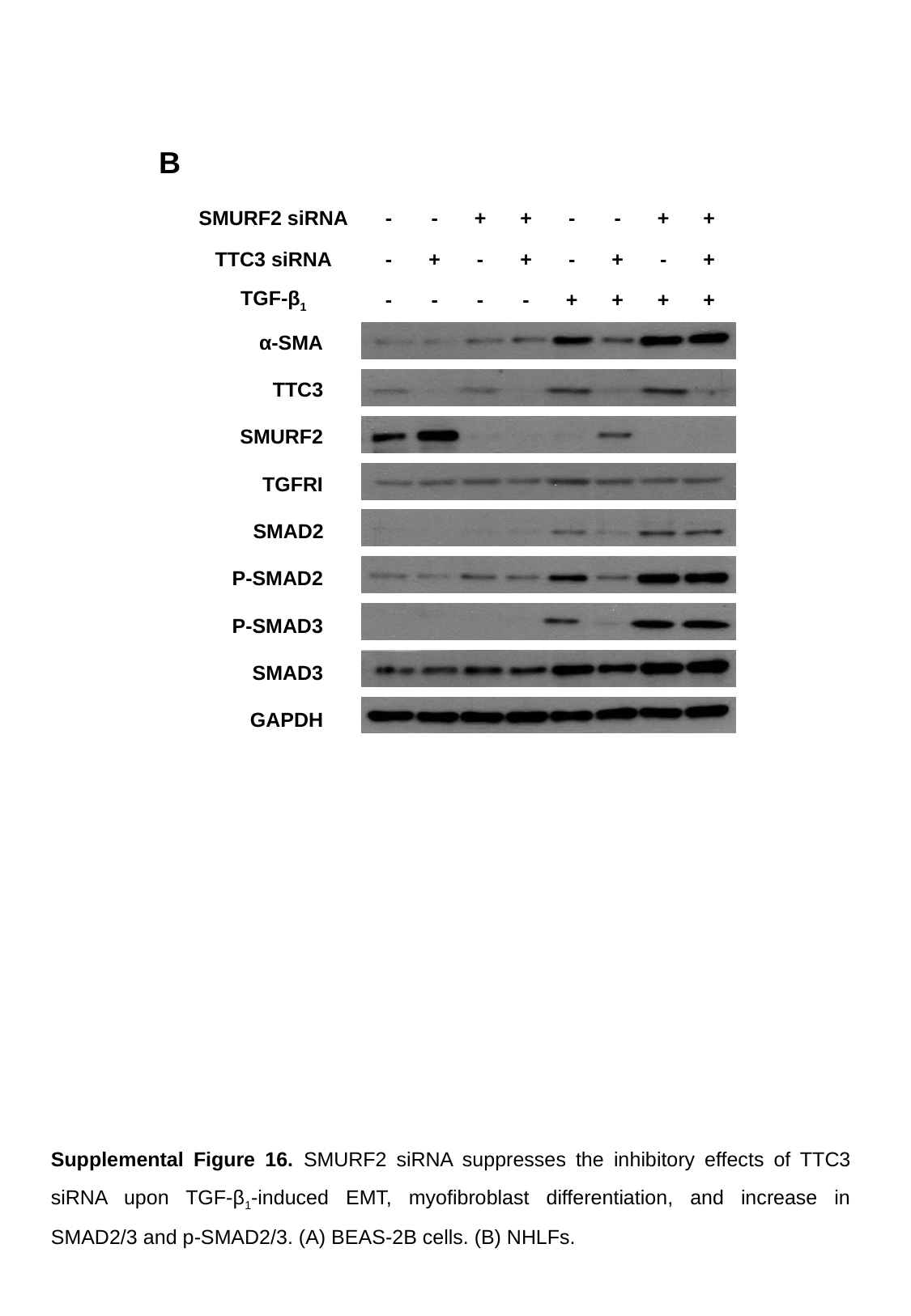

B
| SMURF2 siRNA | - | - | + | + | - | - | + | + |
| --- | --- | --- | --- | --- | --- | --- | --- | --- |
| TTC3 siRNA | - | + | - | + | - | + | - | + |
| TGF-β1 | - | - | - | - | + | + | + | + |
α-SMA
TTC3
SMURF2
TGFRI
SMAD2
P-SMAD2
P-SMAD3
SMAD3
GAPDH
Supplemental Figure 16. SMURF2 siRNA suppresses the inhibitory effects of TTC3 siRNA upon TGF-β1-induced EMT, myofibroblast differentiation, and increase in SMAD2/3 and p-SMAD2/3. (A) BEAS-2B cells. (B) NHLFs.

## Slide 20
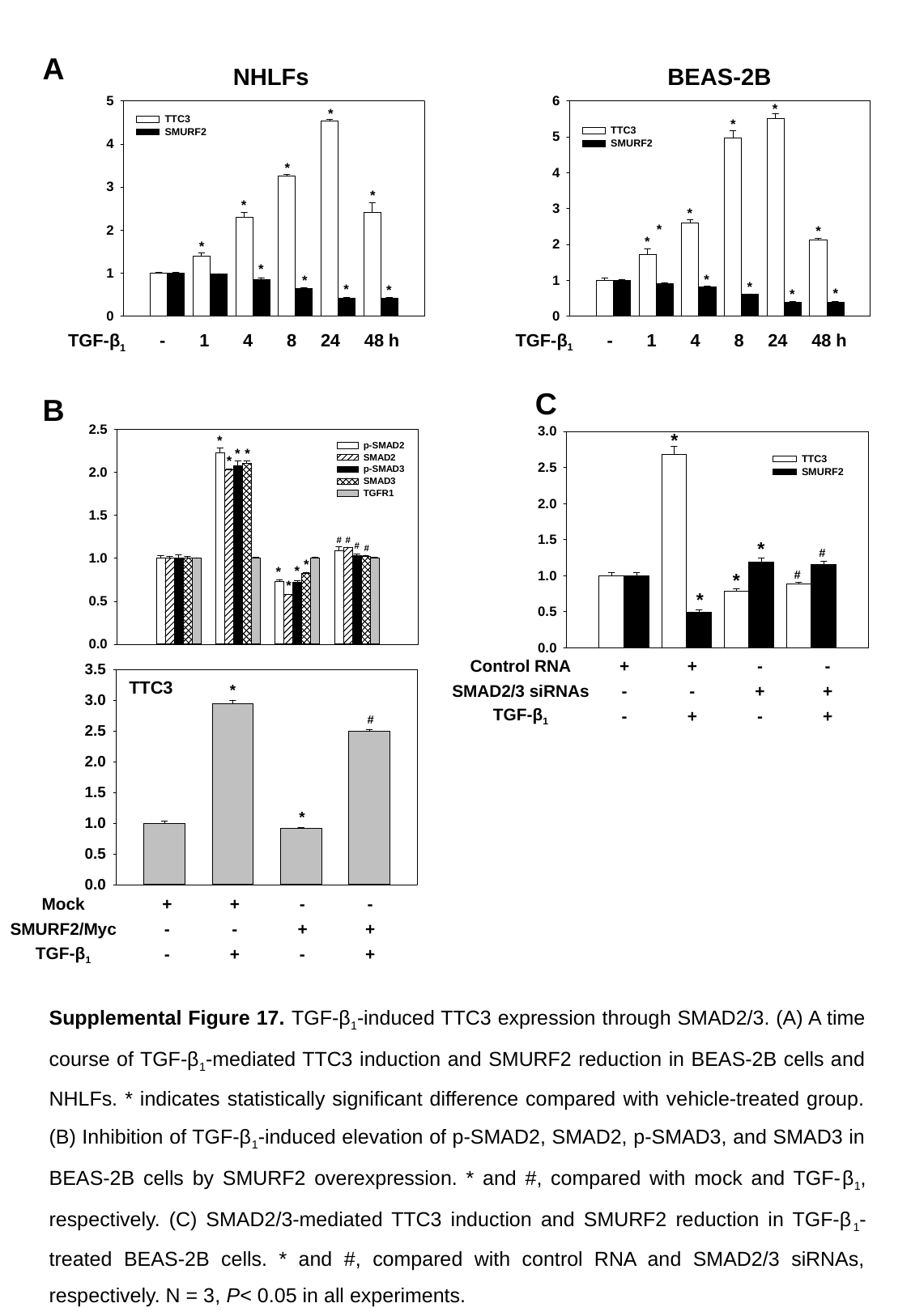

A
BEAS-2B
NHLFs
| TGF-β1 - 1 4 8 24 48 h |
| --- |
| TGF-β1 - 1 4 8 24 48 h |
| --- |
C
B
| Control RNA | + | + | - | - |
| --- | --- | --- | --- | --- |
| SMAD2/3 siRNAs | - | - | + | + |
| TGF-β1 | - | + | - | + |
TTC3
| Mock | + | + | - | - |
| --- | --- | --- | --- | --- |
| SMURF2/Myc | - | - | + | + |
| TGF-β1 | - | + | - | + |
Supplemental Figure 17. TGF-β1-induced TTC3 expression through SMAD2/3. (A) A time course of TGF-β1-mediated TTC3 induction and SMURF2 reduction in BEAS-2B cells and NHLFs. * indicates statistically significant difference compared with vehicle-treated group. (B) Inhibition of TGF-β1-induced elevation of p-SMAD2, SMAD2, p-SMAD3, and SMAD3 in BEAS-2B cells by SMURF2 overexpression. * and #, compared with mock and TGF-β1, respectively. (C) SMAD2/3-mediated TTC3 induction and SMURF2 reduction in TGF-β1-treated BEAS-2B cells. * and #, compared with control RNA and SMAD2/3 siRNAs, respectively. N = 3, P< 0.05 in all experiments.

## Slide 21
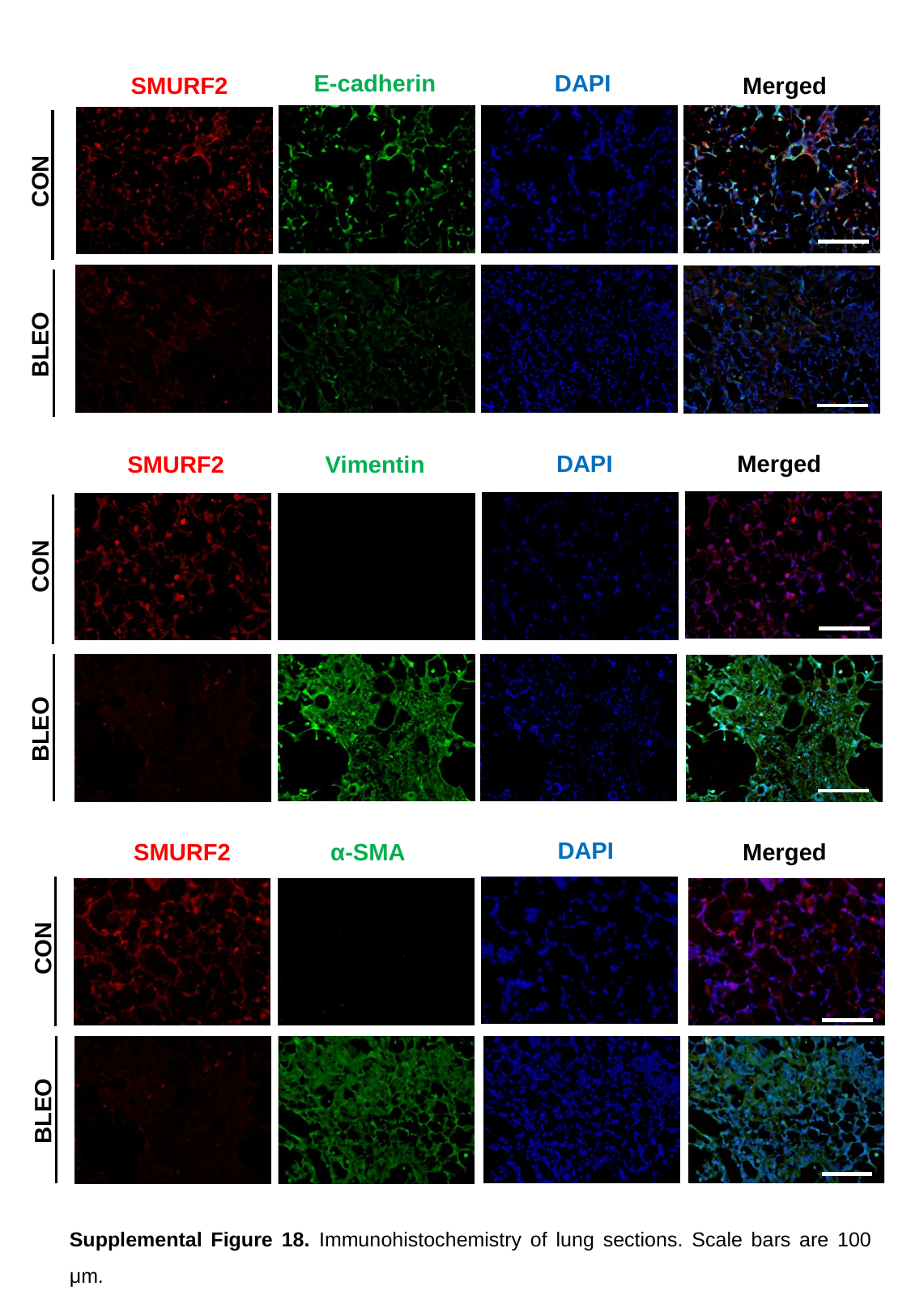

E-cadherin
DAPI
Merged
SMURF2
CON
BLEO
DAPI
Merged
SMURF2
Vimentin
CON
BLEO
DAPI
Merged
SMURF2
α-SMA
CON
BLEO
Supplemental Figure 18. Immunohistochemistry of lung sections. Scale bars are 100 μm.

## Slide 22
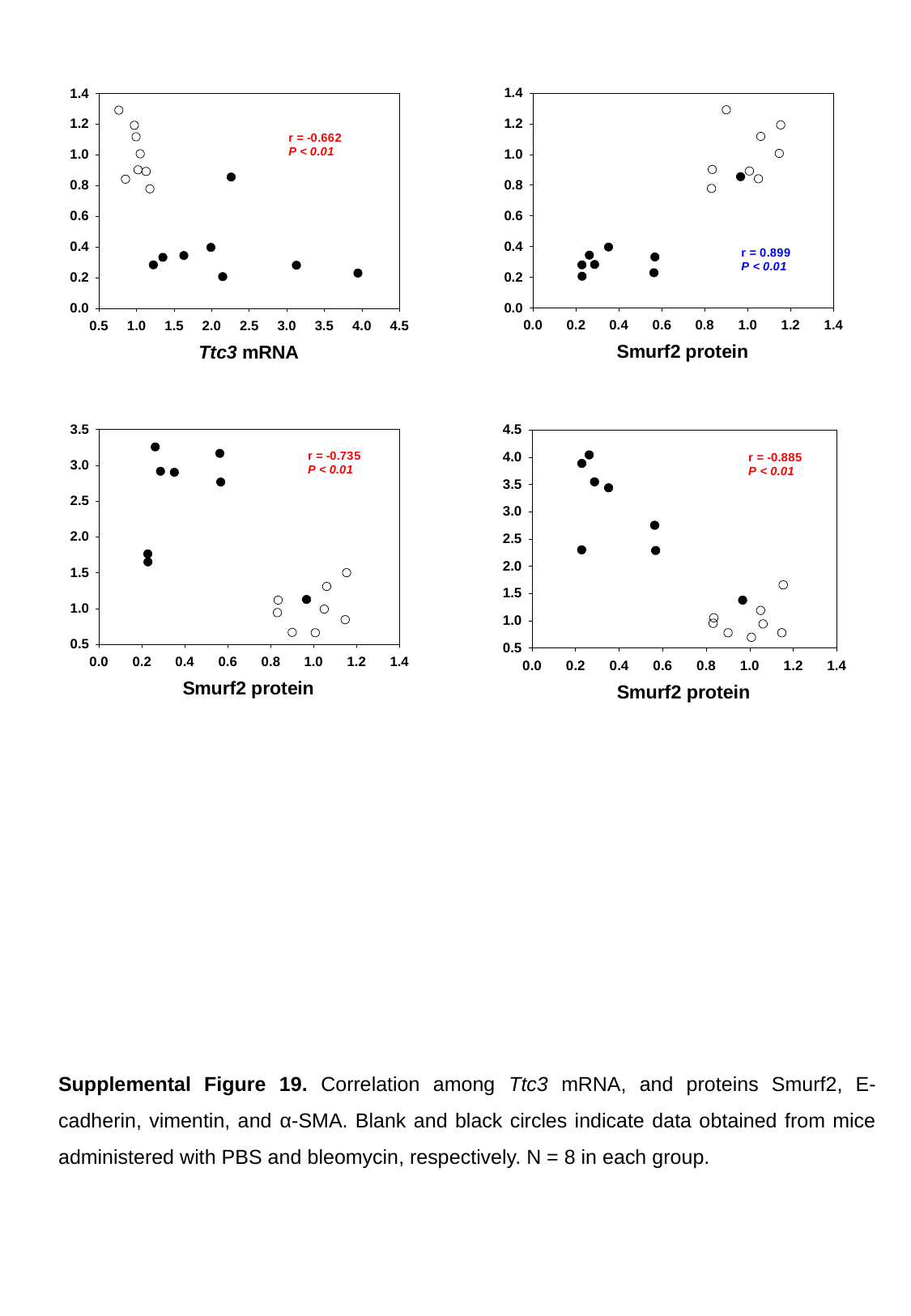

Supplemental Figure 19. Correlation among Ttc3 mRNA, and proteins Smurf2, E-cadherin, vimentin, and α-SMA. Blank and black circles indicate data obtained from mice administered with PBS and bleomycin, respectively. N = 8 in each group.

## Slide 23
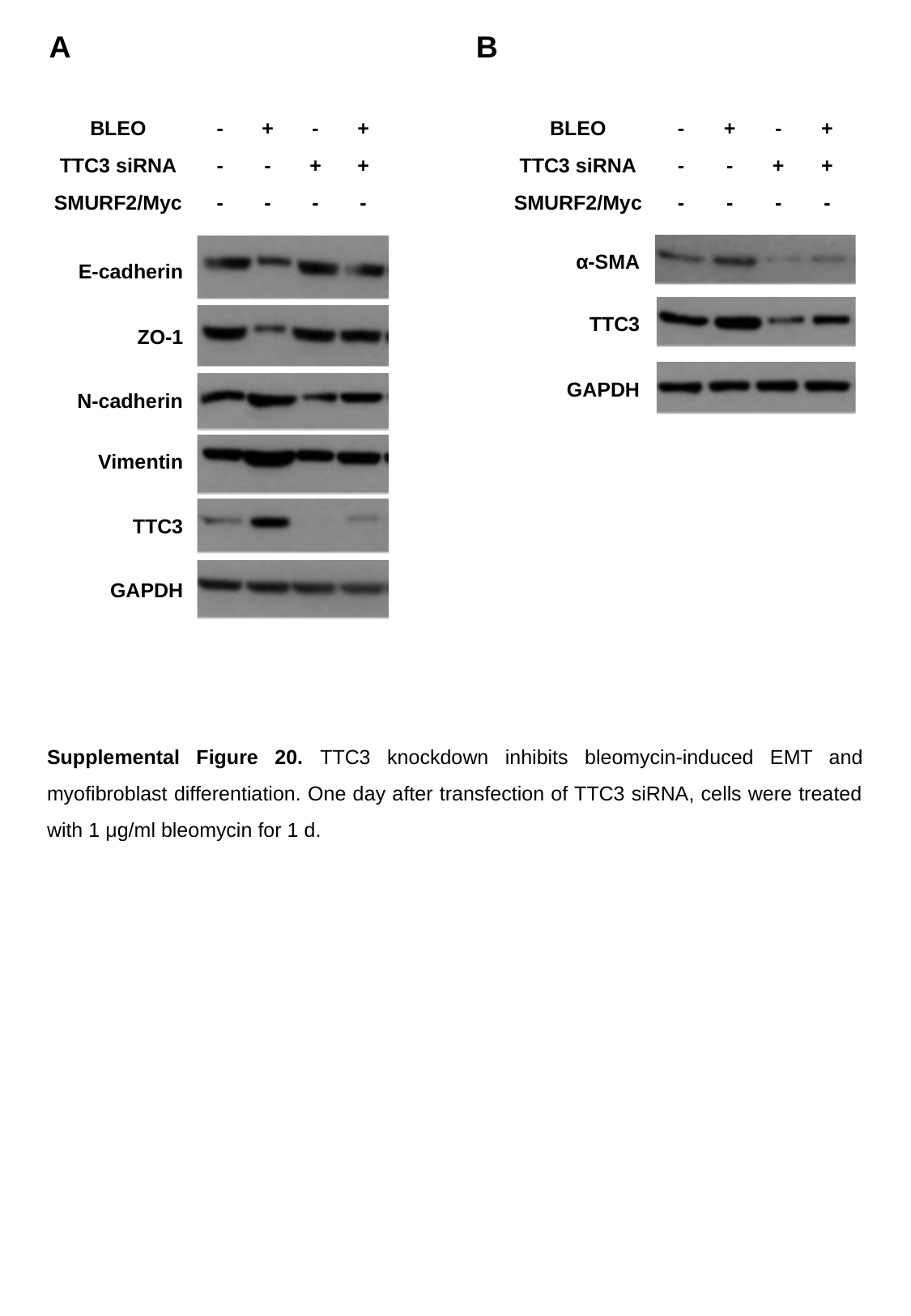

A
B
| BLEO | - | + | - | + |
| --- | --- | --- | --- | --- |
| TTC3 siRNA | - | - | + | + |
| SMURF2/Myc | - | - | - | - |
| BLEO | - | + | - | + |
| --- | --- | --- | --- | --- |
| TTC3 siRNA | - | - | + | + |
| SMURF2/Myc | - | - | - | - |
α-SMA
E-cadherin
TTC3
ZO-1
GAPDH
N-cadherin
Vimentin
TTC3
GAPDH
Supplemental Figure 20. TTC3 knockdown inhibits bleomycin-induced EMT and myofibroblast differentiation. One day after transfection of TTC3 siRNA, cells were treated with 1 μg/ml bleomycin for 1 d.

## Slide 24
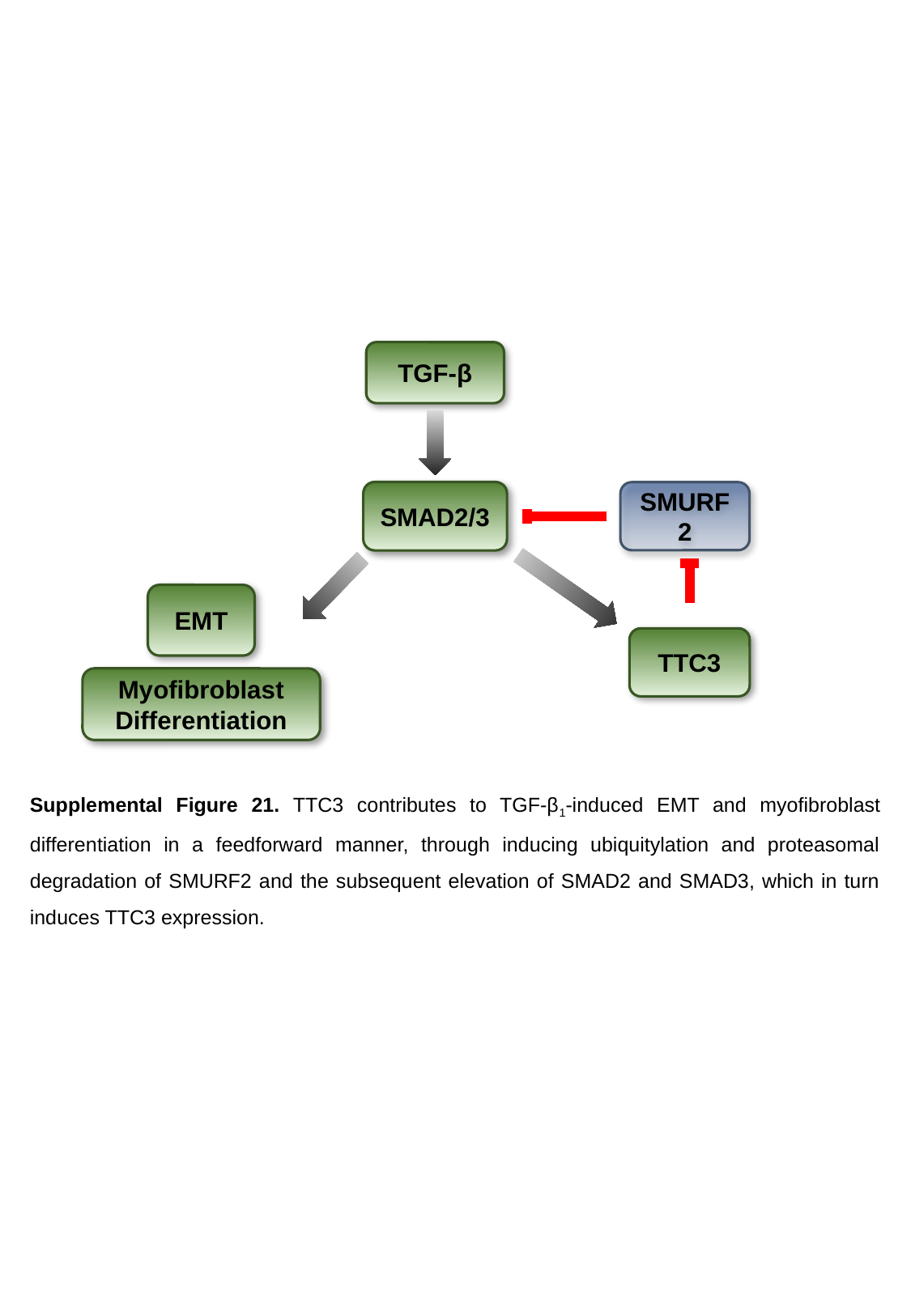

TGF-β
SMAD2/3
SMURF2
EMT
Myofibroblast
Differentiation
TTC3
Supplemental Figure 21. TTC3 contributes to TGF-β1-induced EMT and myofibroblast differentiation in a feedforward manner, through inducing ubiquitylation and proteasomal degradation of SMURF2 and the subsequent elevation of SMAD2 and SMAD3, which in turn induces TTC3 expression.
